# Porous Metals Formed by Leaching Mn–Ni Alloys

**DOI:** 10.1021/acsomega.5c13660

**Published:** 2026-04-27

**Authors:** Thomas A. Manz, David R. Gaskell, Kevin P. Trumble, Zhufang Liu, David Roberts, Carl Hager, Sourav K. Sengupta, Theodore A. Koch, W. Nicholas Delgass

**Affiliations:** † School of Chemical Engineering, 311308 Purdue University, West Lafayette, Indiana 47907, United States; ‡ School of Materials Engineering, Purdue University, West Lafayette, Indiana 47907, United States; § Electron Microscopy Center, Purdue University, West Lafayette, Indiana 47907, United States; ∥ 4156 DuPont Corporation, Wilmington, Delaware 19803, United States

## Abstract

Several Mn–Ni alloys having more than 50 at. % Mn were prepared, and their phase microstructures were examined using optical microscopy, electron microprobe analysis, and X-ray diffraction. These alloys were leached in concentrated acetic acid aqueous solutions. The resulting nanoporous materials were characterized using optical microscopy, element composition analysis, and liquid nitrogen physisorption. Three alloy compositions were studied in detail. The first alloy containing ∼57 at. % Mn was only leachable near the surface. The second alloy containing ∼86 at. % Mn was readily leachable in concentrated acetic acid aqueous solution. Butyronitrile hydrogen reaction was performed over the resulting porous catalyst. The third alloy containing ∼73 at. % Mn was strong, nonbrittle, and did not deteriorate over more than 25+ years of observation. When this alloy was leached in concentrated acetic acid aqueous solution for up to 7 days, a nanoporous brick-like microstructure was produced that is extremely strong, nonbrittle, and lightweight with a low overall density. This material should find uses in applications that require strong, lightweight materials. When leached for 14 days, enough of the manganese atoms were removed that the microstructure had transitioned to a powdery macroporous material.

## Graphical abstract

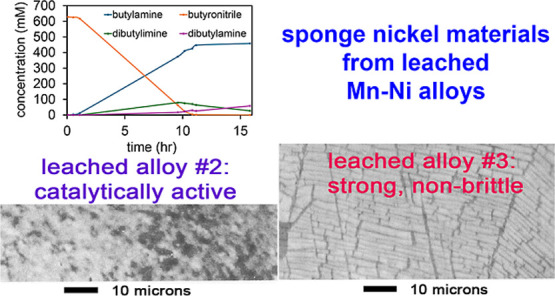

## 1 Introduction

### 1.1 Preparing Nanoporous Metals Using Selective Leaching in Aqueous Solutions

One strategy to prepare nanoporous metals is to create an alloy comprised of at least two metallic elements and then leach out one of the metallic elements leaving the other metallic element behind.^1^ During leaching, one of the alloy’s metallic elements should be oxidized and dissolved in aqueous solution while the other metallic element in the alloy should not be oxidized under such conditions. Hence, there should be a large difference in electrochemical oxidation potentials between the two metallic elements comprising the alloy.

The aqueous solution that performs this leaching is typically either an acidic solution or a basic solution. During this leaching process, hydrogen gas is released. Representing the nonleachable metallic element as “A” and the leachable metallic element as “B”, the following chemical reactions are indicated

1 $$\begin{array}{l} acidic\;solution:\\ \begin{array}{l} AB_{x}\\ \left(solid\right) \end{array}+\begin{array}{l} n\left(x-y\right)H^{+}\\ \left(aqueous\right) \end{array}\to \begin{array}{l} AB_{y}\\ \left(solid\right) \end{array}+\begin{array}{l} \left(x-y\right)B^{n+}\\ \left(aqueous\right) \end{array}+\begin{array}{l} 0.5n\left(x-y\right)H_{2}\\ \left(gas\right) \end{array} \end{array}$$

2 $$\begin{array}{l} basic\;solution:\\ \begin{array}{l} AB_{x}\\ \left(solid\right) \end{array}+\begin{array}{l} n\left(x-y\right)H_{2}O\\ \left(aqueous\right) \end{array}\to \begin{array}{l} AB_{y}\\ \left(solid\right) \end{array}+\begin{array}{l} \left(x-y\right)B^{n+}\\ \left(aqueous\right) \end{array}+\begin{array}{l} 0.5n\left(x-y\right)H_{2}\\ \left(gas\right) \end{array}+\begin{array}{l} n\left(x-y\right)OH^{-}\\ \left(aqueous\right) \end{array} \end{array}$$

where *x* > *y*.

In basic solutions, some of the hydroxide ions are typically complexed with the leached cation. For example, one of the aqueous products of leaching aluminum in basic sodium hydroxide solutions is [Al­(OH)_4_]^−^

3 $$\begin{array}{l} aluminum\;leaching\;in\;basic\;sodium\;hydroxide\;solutions:\\ \begin{array}{l} AAl_{x}\\ \left(solid\right) \end{array}+\begin{array}{l} 3\left(x-y\right)H_{2}O\\ \left(aqueous\right) \end{array}+\begin{array}{l} \left(x-y\right)NaOH\\ \left(aqueous\right) \end{array}\to \begin{array}{l} AAl_{y}\\ \left(solid\right) \end{array}+\begin{array}{l} \left(x-y\right){\left[Al{\left(OH\right)}_{4}\right]}^{-1}\\ \left(aqueous\right) \end{array}+\begin{array}{l} 1.5\left(x-y\right)H_{2}\\ \left(gas\right) \end{array}+\begin{array}{l} \left(x-y\right)Na^{+1}\\ \left(aqueous\right) \end{array} \end{array}$$

Al^3+^ is soluble in strong bases, because it forms the soluble [Al­(OH)_4_]^−^ ion instead of Al­(OH)_3_.

To leach element B but not element A, metallic element B should have a higher oxidation potential than H_2_, while metallic element A should have a lower oxidation potential than H_2_. Notably, the oxidation potential of H_2_ depends on the aqueous solution’s pH value. At pH = 0, the oxidation potential of H_2_ is 0.00 V and corresponds to the half reaction H_2_ (1 atm) → 2H^+^ (1 molar) + 2 e^–^. At pH = 14, the oxidation potential of H_2_ is 0.83 V and corresponds to the half reaction H_2_ (1 atm) + 2 OH^–^ (1 molar) → 2H_2_O + 2 e^–^.^2^ For intermediate pH values, the correct oxidation potential value can be computed using the Nernst equation.^2^

There is a long and rich history of preparing sponge nickel, cobalt, and copper materials in this manner. Murray Raney pioneered the development of these material types.^1,3–7^ Many diverse metal combinations have been investigated in the preparation of sponge metal materials.^5,7–13^ Bekassy, Petro, and co-workers published a multipart series of journal articles that studied sponge nickel materials prepared by leaching Ni–Al, Ni–Si, Ni–Al–Si, Ni–Mg, and Ni–Zn alloys.^9,14–21^

An “unpromoted” Raney nickel catalyst prepared by leaching a Ni–Al alloy (50 wt % Ni, 50 wt % Al) in concentrated sodium hydroxide aqueous solution (for 1 h at 90 °C) can be taken as a sort of “reference” catalyst against which to compare the reaction performance of other sponge nickel catalysts. (Raney­(r) Ni is a registered trademark of W.R. Grace & Co.) Several experimentally measured properties of this Raney nickel unpromoted Ni–Al catalyst were reported in prior work.^11,22,23^

In dealloyed materials, a more reactive chemical element is selectively removed from the alloy to leave behind a nanoporous framework enriched in the less reactive chemical element. Dealloyed materials have found widespread applications and research interest as lightweight materials for various structural material, sensing, electrolytic, ultracapacitor, and catalytic applications.^24–28^ Both precious^29,30^ and nonprecious^31,32^ metals have been the subjects of dealloying research. Dealloying is also important in some corrosion reactions.^33,34^ Here, we show that dealloying Mn–Ni alloys can be used to produce catalytic materials (see Section 3.2.4 below) and lightweight structural materials (see Section 3.3.1 and Section 4 below).

In this work, we studied the leachability of binary Mn–Ni alloys in aqueous solutions to produce sponge nickel materials. Our choice of using manganese as the leachable component for making new sponge nickel materials was motivated by a close examination of the standard electrochemical potentials in aqueous solution^2^ and the binary alloy phase diagrams^35,36^ of various chemical elements. Although Al–Ni–Mn alloys have been investigated previously,^12,37^ research on the leachability of binary Mn–Ni alloys is scarce. Hakamada et al. investigated the electrochemical dealloying of NiMn alloys driven by applying an external voltage in which the NiMn alloy functions as one of the electrodes in an electrochemical cell.^38–40^

The relevant half-reactions for leaching NiMn alloys are^2^

4 $$\begin{array}{l} Mn\\ \left(solid\right) \end{array}+\begin{array}{l} 2H^{+}\\ \left(aqueous\right) \end{array}\to \begin{array}{l} Mn^{2+}\\ \left(aqueous\right) \end{array}+\begin{array}{l} H_{2}\\ \left(gas\right) \end{array},E^{0}=1.18volt,{,E}^{pH4}\approx 0.94\;volt$$

5 $$\begin{array}{l} Ni\\ \left(solid\right) \end{array}+\begin{array}{l} 2H^{+}\\ \left(aqueous\right) \end{array}\to \begin{array}{l} Ni^{2+}\\ \left(aqueous\right) \end{array}+\begin{array}{l} H_{2}\\ \left(gas\right) \end{array},E^{0}=0.236volt,{,E}^{pH4}\approx 0.0\;volt$$

To prevent nickel atoms from being oxidized (i.e., leached away) the pH of the aqueous solution should be above approximately 4. In aqueous solutions, Mn is oxidized by H^+^ or H_2_O at all pH values. Mn leaching should be performed in acidic solutions to produce aqueous Mn^2+^ ions instead of precipitating Mn­(OH)_2_ or MnO in basic or neutral solutions. In acidic solutions, spontaneous oxidation of Mn^2+^ to Mn^3+^ or higher oxidation states is not favored.^2^ Putting this altogether, MnNi alloys should be leached in aqueous solutions having pH values slightly higher than ∼4 (i.e., weakly acidic solutions) but lower than 7 (neutral solutions).

### 1.2 The Mn–Ni Phase Diagram

The Mn–Ni phase diagram is relatively complicated due to the large number of phases present. Figure 1 (from ref 41), Figure 2 (from ref 42), and Figure 3 (from ref 43) reproduce Mn–Ni phase diagrams constructed by different authors. These three phase diagrams have some aspects of agreement, but they also have some key differences. All three phase diagrams show that pure manganese exists in four allotropic forms: α, β, δ, and γ. The δ form is only stable above 1138 °C and is not relevant to this work. All three phase diagrams show a miscible liquid phase at high temperatures which cools to form a (γMn, Ni) phase for all compositions >10% Ni. All three phase diagrams contain a MnNi_3_ intermetallic phase and at least two different intermetallic phases having MnNi composition. However, there is some disagreement among these phase diagrams on whether MnNi_2_ and NiMn_2_ intermetallic phases exists. This indicates that there are some aspects of the Mn–Ni phase diagram which are still not yet understood. Some unusual phases in the Mn–Ni system were discovered that are still not fully understood.^44,45^

**Figure 1:**
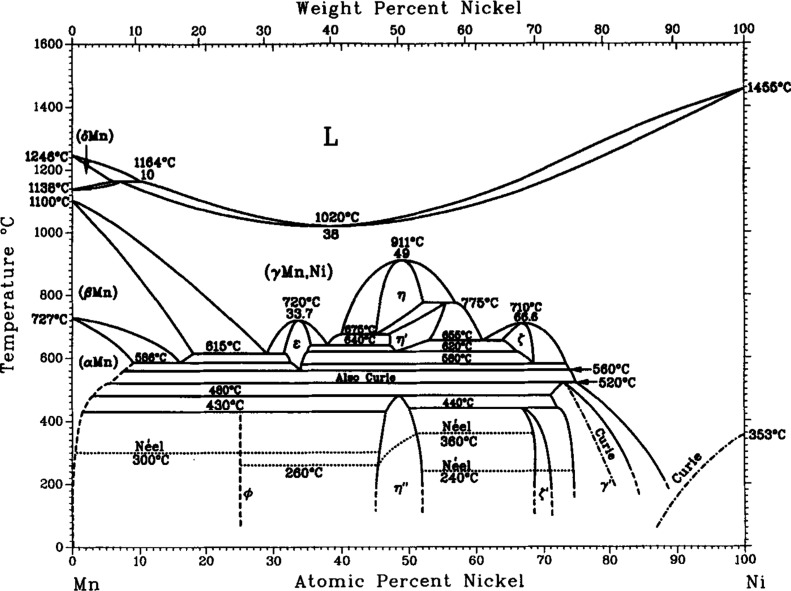
Mn–Ni phase diagram according to Gokcen 1991 (Copyright 1991 Springer. Reproduced with permission from Gokcen, *J. Phase Equil.* **1991**, 12, 313–321.).

**Figure 2:**
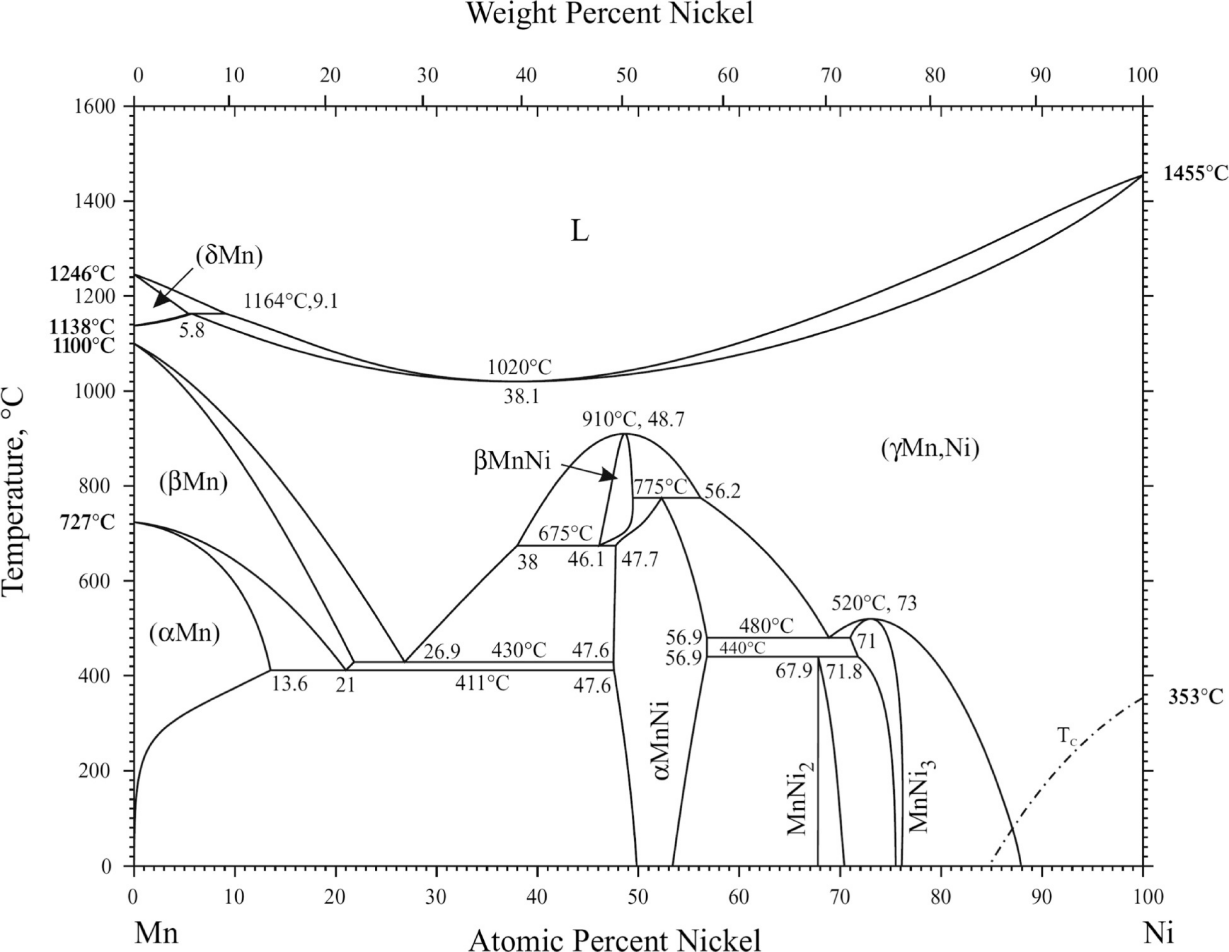
Mn–Ni phase diagram according to Okamoto 2007 (Copyright 2007 Springer. Reproduced with permission from Okamoto, *J. Phase Equilib. Diffus.* **2007**, 28, 406–407.).

**Figure 3:**
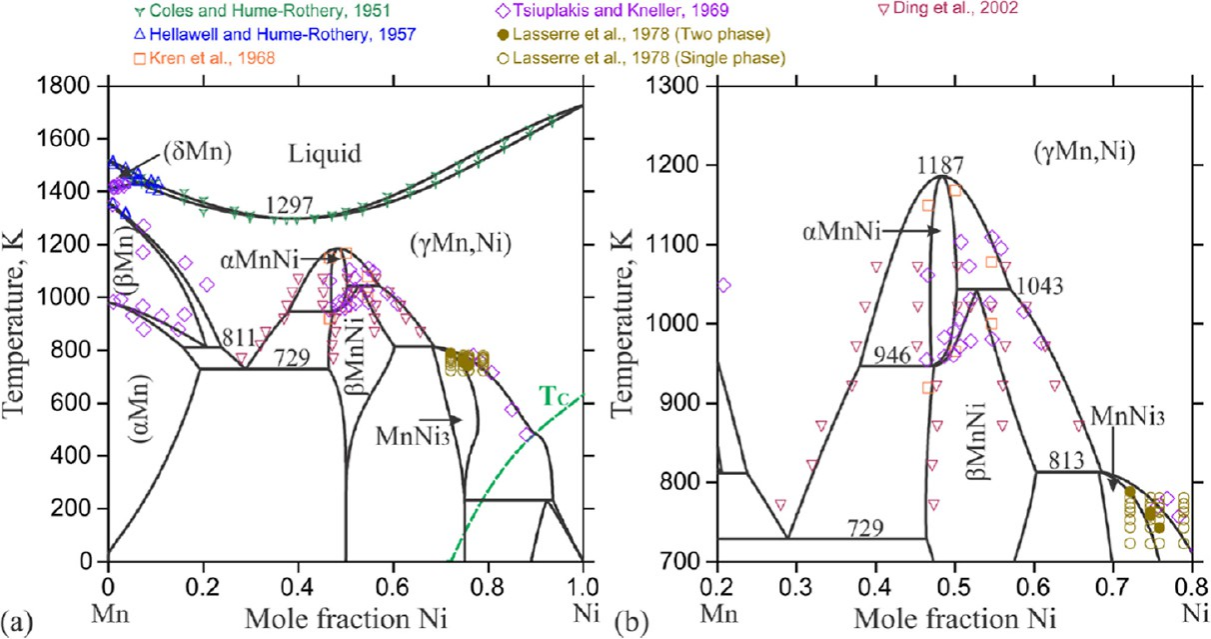
(a) The calculated Mn–Ni phase diagram according to Hao et al. 2024. Symbols denote experimental data from refs 45–50 The Curie temperature is denoted by the green dashed line. (b) The partial Mn–Ni phase diagram demonstrates the phase equilibria among the ordered phases and disordered (γMn,Ni). (Copyright 2024 Springer. Reproduced with permission from Hao et al., *J. Phase Equilib. Diffus.* **2024**, 45, 1182–1193.).

Different authors sometimes refer to the same intermetallic phase by different names. In this article, we adopt the naming convention used by Okamoto 2007 as shown in Table 1 and Figure 2.

**Table 1:** Mn–Ni Crystal Structure Data. Copyright 2007 Springer^a^

phase	composition, at.% Ni	pearson symbol	spacegroup		prototype
(δMn)	0–5.5	cI2	Im3m	A2	W
(γMn, Ni)	0–100	cF4	Fm3m	A1	Cu
(βMn)	0–21.8	cP20	*P*4_1_32	A13	βMn
(αMn)	0–13.6	cI58	I43m	A12	αMn
βMnNi	46.1–49.5	cP2	*Pm* 3m	B2	CsCl
αMnNi	47.6–56.9	tP4	*P*4/*mmm*	L1_0_	AuCu
MnNi_2_	67.9–70.5				
MnNi_3_	71–76	cP4	*Pm* 3m	L1_2_	AuCu_3_

a Reproduced with permission from Okamoto, *J. Phase Equilib. Diffus.* **2007**, *28*, 406–407.

## 2 Experimental Methods

### 2.1 Alloy Synthesis

#### 2.1.1 Alloy #1: High Nickel Content

The Mn metal was washed in acetic acid to remove oxide. The first alloy was made from a charge of 2.69 g Ni and 5.43 g Mn. This corresponds to a target composition of 66.9 wt % Mn (68.5 at. %). The charge was placed in glass ampule, and glass wool was put into a neck of the glass ampule to reduce evaporation. The glass ampule containing the sample was placed into a silica-glazed porcelain boat and heated in a Lindberg tube furnace. To prevent oxidation, helium flowed through the furnace at 40 mL/min. The charge was heated from 25 to 1280 °C at the rate of 210 °C/h. (The heating and cooling rates could not exceed 250 °C/h because this could potentially crack the furnace tube.) The furnace was held at 1280 °C for 2 h and then cooled slowly. After 6.5 h, the temperature was found to be 360 °C. This corresponds to an average cooling rate of 142 °C/h. When cool, the sample was removed.

Separation of the boat from the alloy was not difficult and the alloy billet remained in one piece of mass 6.00 g. Seventy-four percent of the original mass was recovered. The porcelain boat was somewhat corroded and deformed by the alloy.

A theoretical composition of 56.9 at. % Mn would be calculated based on the assumption that the mass lost was due entirely to Mn vaporization. However, electron microprobe analysis showed that the sample was composed of Mn, Ni, and Si. The silicon came from dissolving part of the glass ampule. The silicon concentration was not explicitly measured with electron microprobe, but it could be estimated as shown in Table 2. Electron microprobe showed the average Mn/Ni ratio was approximately 1.3 ± 0.3.

**Table 2:** Summary of Electron Microprobe Analysis Results on the Unleached Mn–Ni Alloys

	point #	wt %	wt % total	normalized at.%
Mn standard	1	98.60 Mn, 0.00 Ni, 0.015 Si	98.6	99.97 Mn, 0.00 Ni, 0.03 Si
Mn standard	2	97.65 Mn, 0.00 Ni, 0.027 Si	97.7	99.95 Mn, 0.00 Ni, 0.05 Si
Ni standard	1	0.00 Mn, 101.47 Ni, 0.056 Si	101.5	0.00 Mn, 99.88 Ni, 0.12 Si
alloy #1	1^a^	44.44 Mn, 45.34 Ni, (10.2^c^) Si	(100)	41.6 Mn, 39.7 Ni, (18.7^c^) Si
alloy #1	2^a^	44.06 Mn, 45.84 Ni, (10.1^c^) Si	(100)	41.3 Mn, 40.2 Ni, (18.5^c^) Si
alloy #1	3^b^	52.03 Mn, 38.87 Ni, (9.1^c^) Si	(100)	49.0 Mn, 34.2 Ni, (16.8^c^) Si
alloy #1	4^b^	51.62 Mn, 38.68 Ni, (9.7^c^) Si	(100)	48.3 Mn, 33.9 Ni, (17.8^c^) Si
alloy #2	1	84.15 Mn, 15.41 Ni, 1.44 Si	101.0	83.01 Mn, 14.22 Ni, 2.77 Si
alloy #2	2	84.25 Mn, 15.60 Ni, 1.49 Si	101.3	82.79 Mn, 14.34 Ni, 2.87 Si
alloy #3	1^a^	69.65 Mn, 30.46 Ni, 0.18 Si	100.3	70.70 Mn, 28.93 Ni, 0.37 Si
alloy #3	2^a^	68.87 Mn, 29.29 Ni, 0.15 Si	98.3	71.31 Mn, 28.38 Ni, 0.31 Si
alloy #3	3^b^	71.79 Mn, 28.07 Ni, 0.16 Si	100.0	72.98 Mn, 26.70 Ni, 0.32 Si
alloy #3	4^b^	72.89 Mn, 27.15 Ni, 0.09 Si	100.1	74.02 Mn, 25.80 Ni, 0.18 Si

a Lighter phase.

b Darker phase.

c For alloy #1, a large Si peak was observed, but was not directly quantified; for this alloy, the Si percentage was estimated by taking 100% minus the measured Mn and Ni percentages.

#### 2.1.2 Alloy #2: Low Nickel Content

The second alloy was made in manner similar to the first. However, this time a charge of 3.92 g Ni and 24.2 g Mn was used. This corresponds to a target composition of 86.l wt % Mn (86.9 at. %). The charge was heated to 1280 °C and held at this temperature for one instead of 2 h. The final billet remained in several large pieces and had a total mass of 25.0 g. Eighty-nine percent of the original mass was recovered.

A theoretical composition of 85.2 at. % Mn was calculated based on the assumption that the mass lost was due entirely to Mn vaporization or oxidation. The alloy composition was estimated to be the average of this value and the initial composition (86.9 at. %) with a standard deviation of one-half the difference (86.1 ± 0.5 at. %).

When the alloy was analyzed by electron microprobe, a silica peak was present, suggesting that the melt dissolved some of the boat glazing. To prevent this problem, Alundum boats were ordered and used in the preparation of alloy #3. (Alundum­(r) is a registered trademark of Saint-Gobain.) These corrosion-resistant combustion boats are made of fused aluminum oxide grains.

#### 2.1.3 Alloy #3: Medium Nickel Content

A third alloy was prepared from 5.42 g Ni and 14.30 g Mn with a target composition of 72.5 wt % Mn (73.6 at. %). The temperature program was the same used for alloy #2, except the alloy was heated to 1260 °C instead of 1280 °C. An Alundum boat was used to avoid reaction between the melt and container. The alloy billet was removed without breaking the boat and found to have a mass of 18.8 g. More than 95% of the initial mass was recovered.

Using the final weight to verify composition gives 72.3 at. % Mn. The alloy composition was estimated to be the average of the target and calculated compositions with a standard deviation of one-half the difference (73.0 ± 0.7 at. %).

### 2.2 Sample Mounting, Electron Microprobe Analysis, and Optical Microscopy

Sample mounting and microscopy were done in the School of Materials Engineering at Purdue University. A diamond abrasive saw was used to cut each initial metal sample to produce a flat surface for mounting. The initial metal sample was clamped into a mechanical arm and lowered onto the saw. A small amount of oil was dripped onto the blade to lubricate it. The slow, steady action of the saw produced a smooth cut.

Each sample was mounted in a 1 in. Bakelite press. The sample was placed face-down in the press and covered with about 1.5 cm of Bakelite powder. The press was turned on and heated to cure the Bakelite.

After mounting in Bakelite, each sample was smoothed by sanding the flat surface successively on 240, 320, 400, and 600 wet grit papers. The sample was rinsed with water and rotated 90 deg between sanding steps. Each step was completed when all of the scratches were removed perpendicular to the sanding direction. After sanding, each sample was polished on a 800 grid wheel, a 4–8 μm diamond wheel, and a 0.05 μm alumina wheel. The sample was gently rotated during polishing. Sample preparation was complete when the surface appeared mirror-like and free of visible scratches.

After polishing as described above, these Bakelite-mounted unleached metal samples were examined using electron microprobe analysis. Electron microprobe analysis measured the elemental composition of extremely small regions of the sample’s surface. This allowed the elemental composition of individual grains and phases to be measured with high precision.

Table 2 summarizes the electron microprobe analysis results. The indications “point 1” and “point 2” represent different locations on the sample. The column labeled “wt % total” is the sum of unnormalized Mn, Ni, and Si weight percentages; this total should be approximately 100%. The column labeled “normalized at.%” is the atomic molar percentages of Mn, Ni, and Si normalized to add up to 100%.

Subsequently, pictures of the microstructure were taken using an optical microscope equipped with a Polaroid camera. Various magnifications (e.g., 32×, 50×, 100×, 200×, 500×, and 1000×) were used to look at the microstructure. As described in Section 3 below, photos of the microstructure were taken before and after various leaching treatments.

### 2.3 Composition Analysis Using Atomic Absorption Spectroscopy

Elemental analysis compositions of the bulk materials were measured using a PerkinElmer 3110 Atomic Absorption Spectrometer. This atomic absorption spectrometer used a hollow-cathode lamp comprising a tungsten anode and a cathode (made from the chemical element to be quantified) sealed in a glass bulb containing an inert gas (e.g., argon) at low pressure. A separate bulb is used for each chemical element that will be quantified. In this technique, the sample to be analyzed is first completely dissolved in a liquid solution. In this work, an approximately 0.2 g sample of the material to be analyzed was completely dissolved in an aqueous mixture of hydrochloric and nitric acids. Once injected into the instrument, the sample solution is flame ionized. Standards were prepared and used as calibration references to convert the spectrometer’s output signal into solution-phase concentration. A measurement of this type was performed for each chemical element to be quantified. The solution-phase concentrations were then converted (using knowledge of the weight of sample dissolved in the solution) into compositions of the original sample material. A more fulsome description of this measurement technique including the double-beam atomic absorption spectrometer schematic, working physical principles and equations, and calibration and use procedures is contained in the Ph.D. dissertation of Thomas-Pryor from the Delgass research group.^51^

### 2.4 Butyronitrile Hydrogenation Reaction for Catalysts Prepared from Mn–Ni Alloy #2

Butyronitrile hydrogenation was carried out at room temperature (20–25 °C) in a stirred batch reactor. Figure 4 shows the reactor setup. An alcohol–water mixture was used as the solvent for the reactions: 50 mL alcohol plus 10 mL water. (For some of these hydrogenation experiments, methanol–water was used as the solvent; however, an unknown reaction with methanol was observed over some of the Mn–Ni materials. So, ethanol–water was used as the solvent in the remaining hydrogenation experiments.) Hydrogen flowed through the reactor and out through a bubbler, and this assured a constant one-atmosphere hydrogen pressure. The custom-built glass reactor was jacketed so that it was cooled by flowing tap water. The electric stirrer had a magnetic drive and was set to operate at 2000 rpm to ensure vigorous mixing. The reactor had a sample port covered with a rubber septum so that samples could be withdrawn from the reactor using a syringe. The glass reactor was clamped to a metal stand using foam rubber covered clamps and placed on shock-absorbing pads to minimize vibrations.

**Figure 4:**
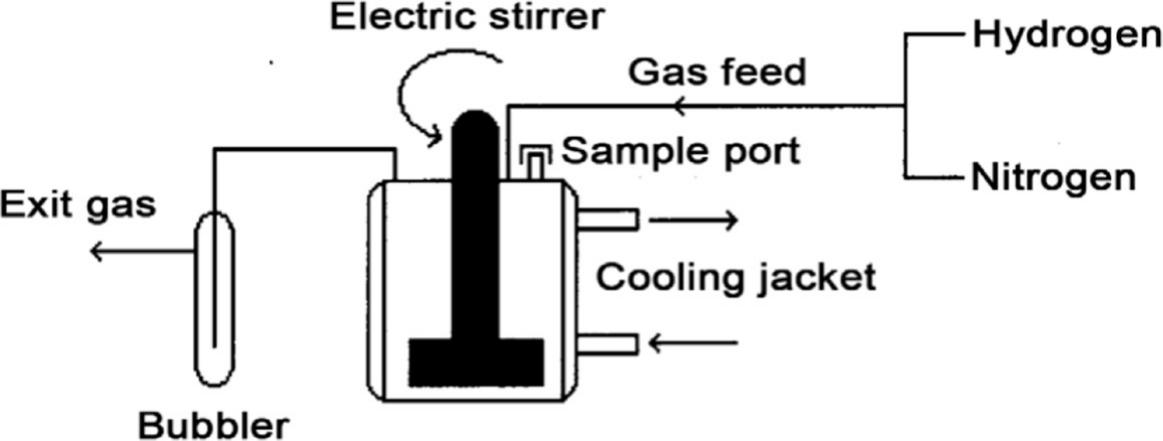
Butyronitrile hydrogenation reactor setup.

A first valve allowed selecting hydrogen or nitrogen as the feed gas, and the flow rate was adjusted using a subsequent variable flow control valve. To avoid mixing hydrogen and oxygen gases in the reactor, the reactor was always first purged with nitrogen gas before starting the hydrogen flow. After the reaction finished, the reactor was always purged with nitrogen gas before opening the reactor. The entire reactor setup was located in a fume hood so that exiting hydrogen gas was quickly dispersed into the flowing air and vented out the fume hood.

During the batch reactions, samples were withdrawn from the glass-jacketed reactor using a glass syringe. These samples were taken at various times during the batch reaction so that concentration versus time plots could be prepared. Reaction mixture composition was measured using gas chromatography as described in ref 52.

Scheme 1 illustrates the reaction scheme for butyronitrile hydrogenation. The prior literature discusses the reaction networks for hydrogenation of butyronitrile^22,53–56^ and related organic nitriles catalyzed by transition metals.^57–61^ Hydrogen adds to the nitrile (in this case, butyronitrile) to form the corresponding primary imine (in this case, butylimine) which is further hydrogenated to the primary amine (in this case, butylamine). Butylimine is a high-energy reactive intermediate that was not directly observed in the reaction products. Butylimine can condense on the catalyst surface to form 1-amino dibutylamine which ejects an ammonia molecule to form dibutylimine (i.e., *N*-butylidene-1-butylamine) which is subsequently hydrogenated to dibutylamine. The nature of the transition metal catalyst controls the ratio of primary to secondary amine produced. For butyronitrile, nickel catalysts preferentially produce the primary amine (i.e., butylamine) with small amounts of secondary amine (i.e., dibutylamine) and practically no tertiary amine (i.e., tributylamine).^22,53,54^ Over some catalysts, further condensation reactions can occur to produce the tertiary amine, but this process is negligible for the catalysts studied here.^53,54^ Palladium and platinum catalysts favor production of the tertiary amine (e.g., tributylamine).^54^

**Scheme 1:**
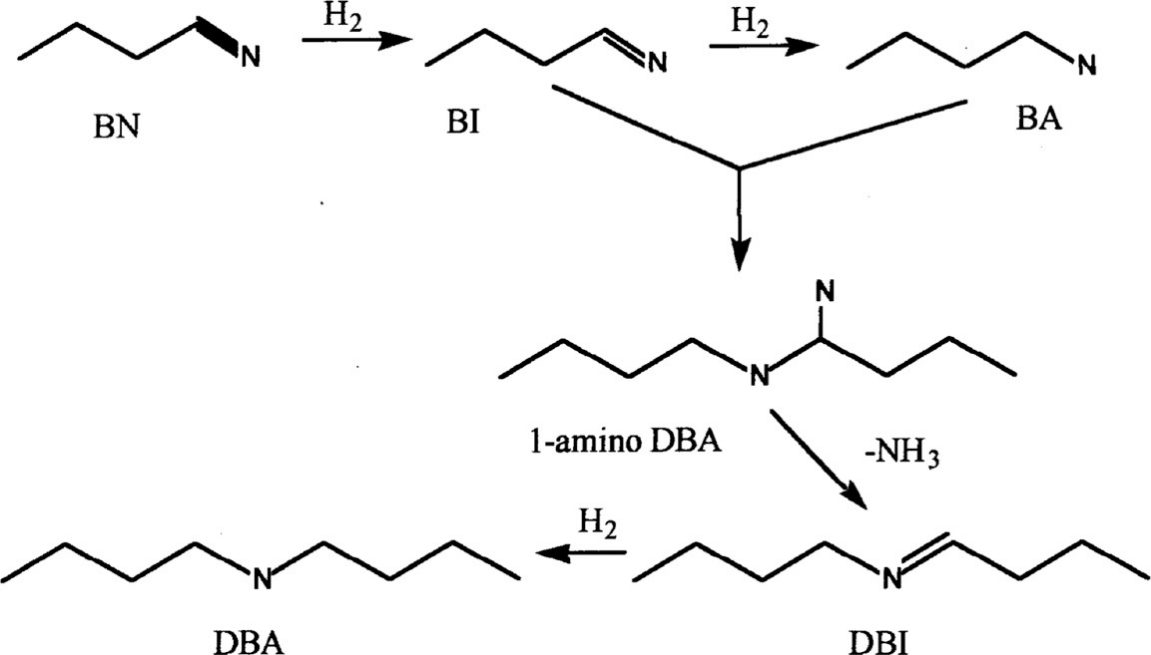
Reaction Scheme for Butyronitrile Hydrogenation^a^

a BN = butyronitrile, BI = butylimine, DBI = dibutylimine, DBA = dibutylamine.

## 3 Results

### 3.1 Alloy # 1: High Nickel Content

#### 3.1.1 Optical Microscopy

Pictures of the microstructure were taken using an optical microscope equipped with a Polaroid camera. The sample preparation and microscopy were done in the School of Materials Engineering at Purdue University. Sample polishing and mounting was done in the manner described in Section 2.2 above.

In optical microscopy, an etchant is often needed to bring out the microstructure. The etchant gives a different shade to each crystal phase. In this study, acids were used to simultaneously leach and etch the surface. As a surface is leached, it becomes darker. The microstructure is apparent because different phases leach at different rates.

Figure 5 shows pictures of the alloy before leaching. No phase structure is visible. The dark areas are voids in the sample caused by shrinkage during solidification.

**Figure 5:**
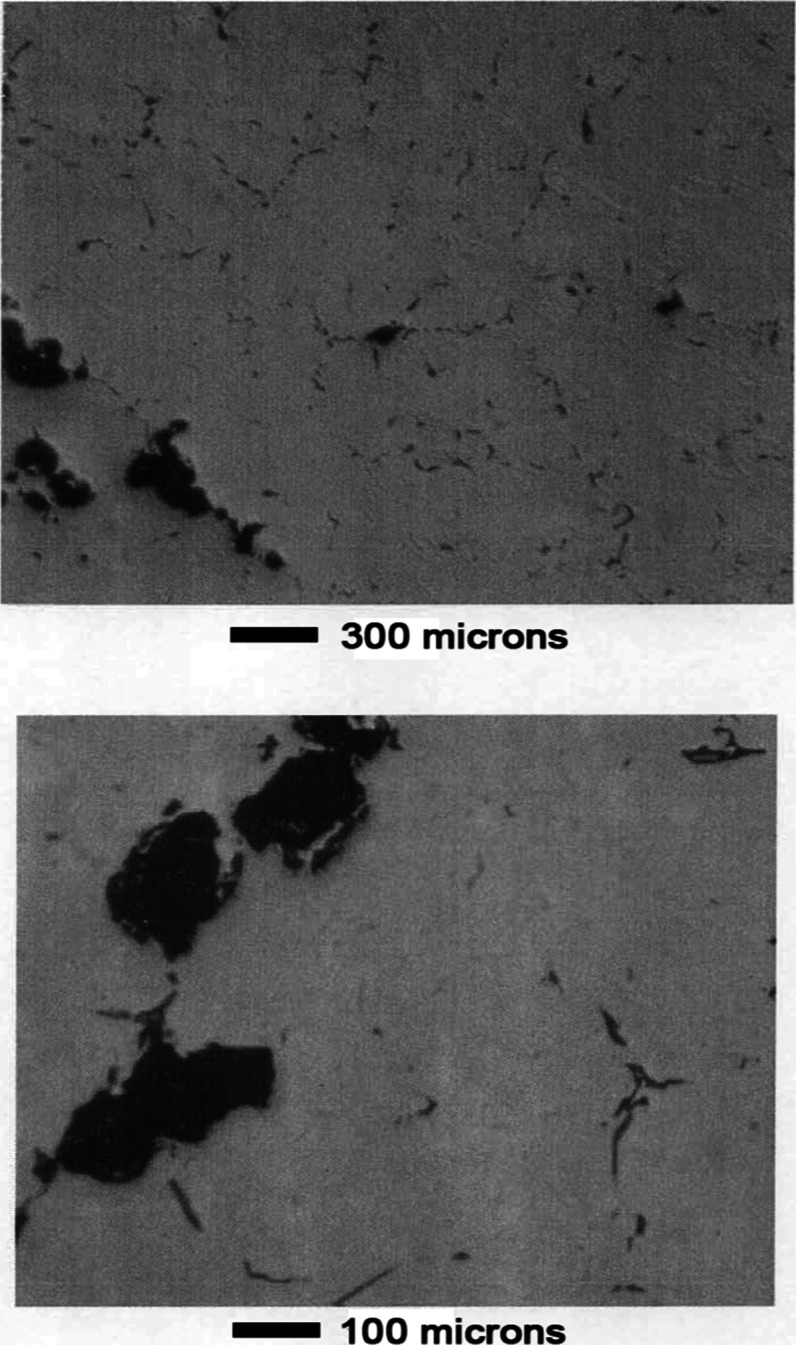
Mn–Ni Alloy #1 before leaching.

The alloy was immersed in 1 wt % nitric acid in ethanol for 115 s. It was then rinsed with ethanol, dried, and examined by optical microscopy, see Figure 6. The phase structure of the alloy became visible. A continuous phase surrounded needle-like grains. Parts of the continuous phase contained no needle-like grains. This gave the appearance of a binary microstructure in which the two regions were: (A) continuous phase and (B.) continuous phase plus needles. Etching occurred primarily at the grain boundaries around the needle-like phase.

**Figure 6:**
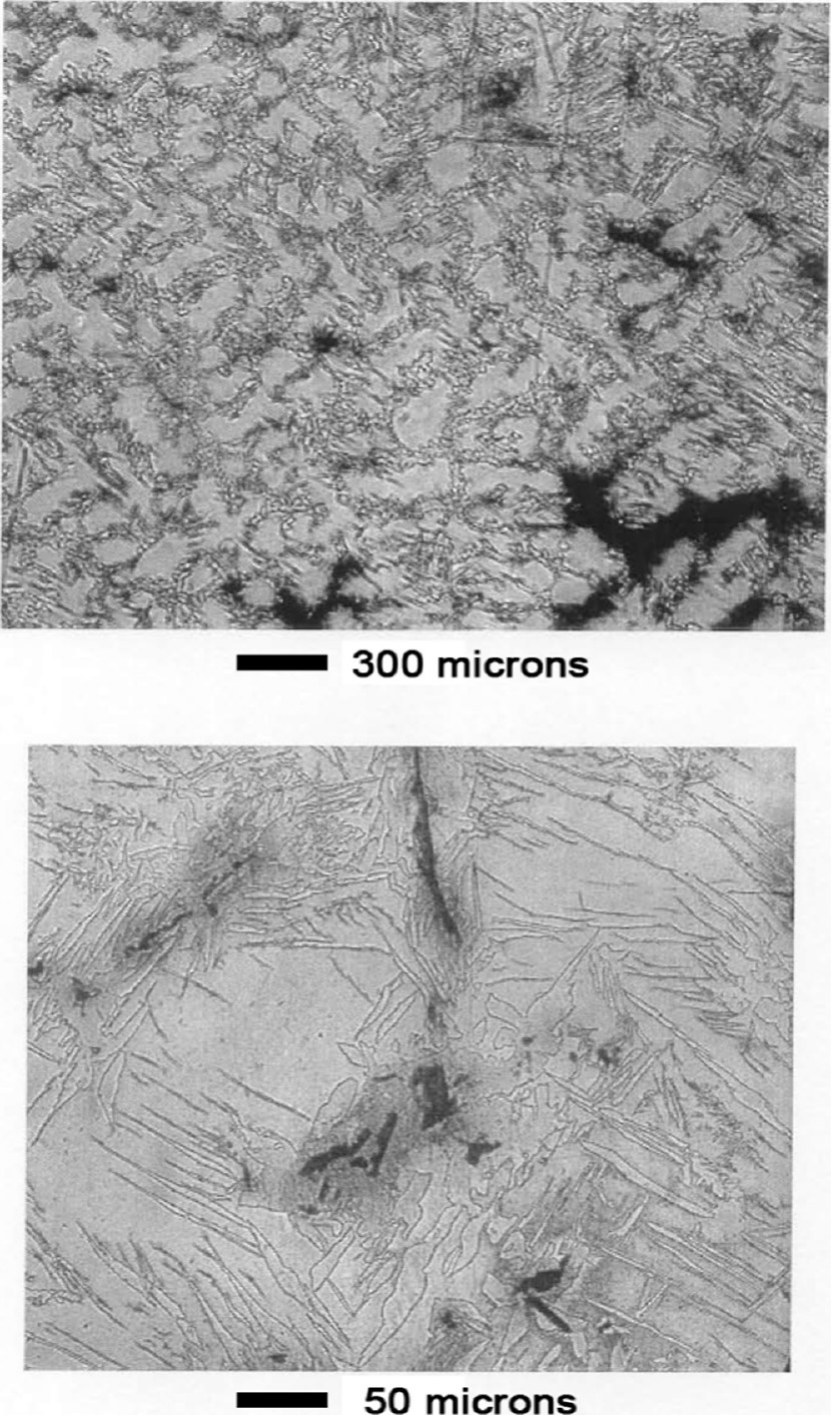
Mn–Ni Alloy #1 after leaching 115 s in 1 wt % nitric acid.

Subsequent leaching attacked first the needle-like phase, Figure 7. Cracks were formed which propagated into the continuous phase, Figure 8. Pits also formed in the surface of the continuous phase, Figure 8. After leaching for a total of 13 min in 1 wt % nitric acid and 17 min in 10 wt % nitric acid, the surface was rough and deeply etched, as shown in Figure 9. Figure 10 compares the surface structure before and after leaching at 500× magnification.

**Figure 7:**
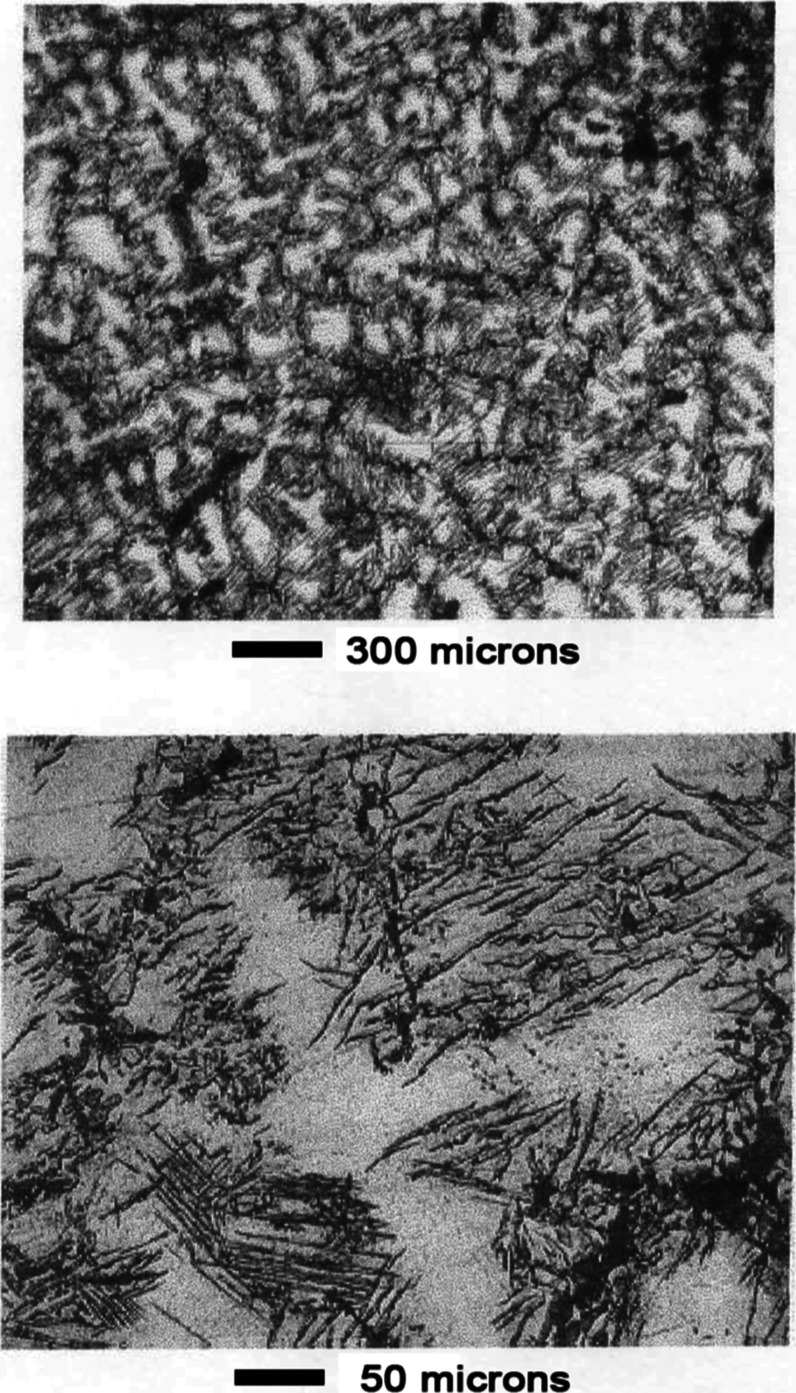
Mn–Ni Alloy #1 after leaching 13 min in 1 wt % nitric acid.

**Figure 8:**
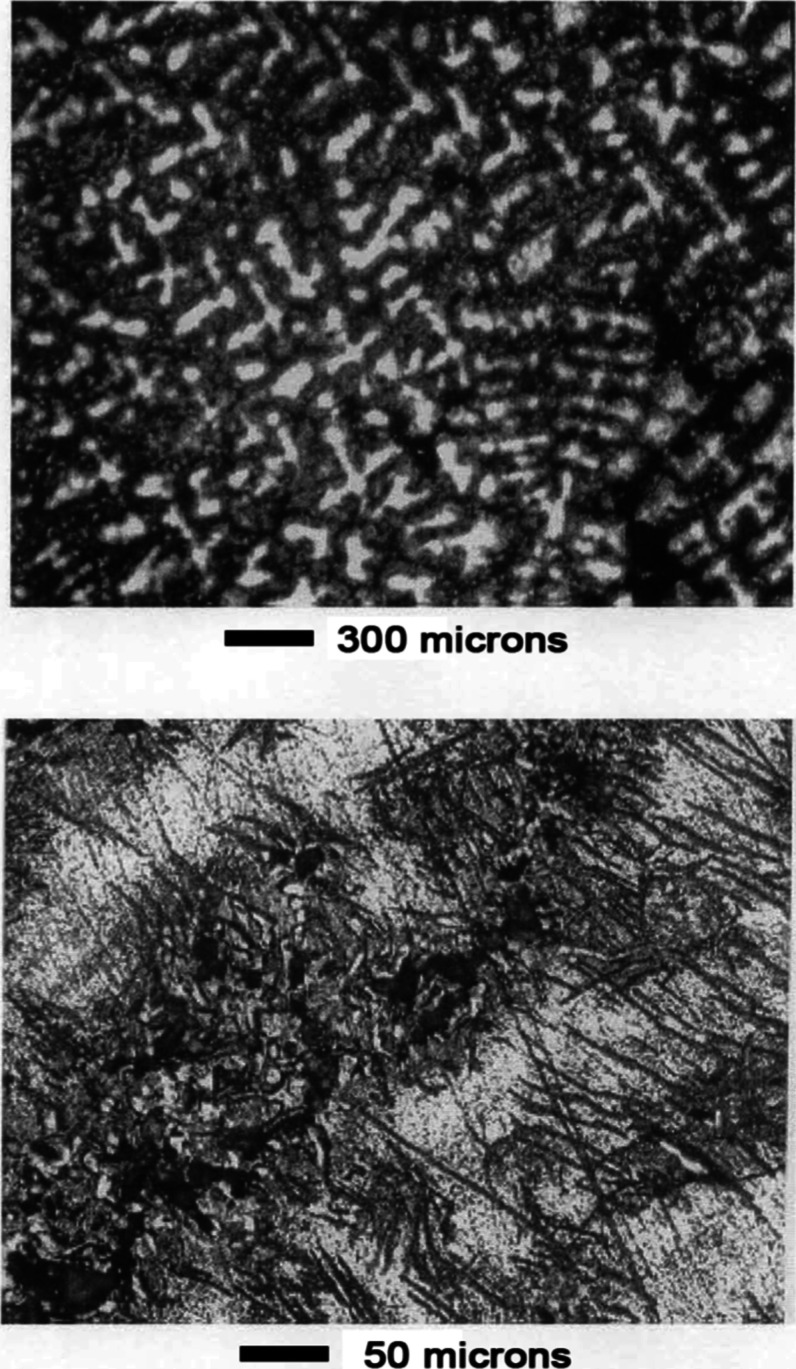
Mn–Ni Alloy #1 after leaching 13 min in 1 wt % nitric acid and 5 min in 10 wt % nitric acid.

**Figure 9:**
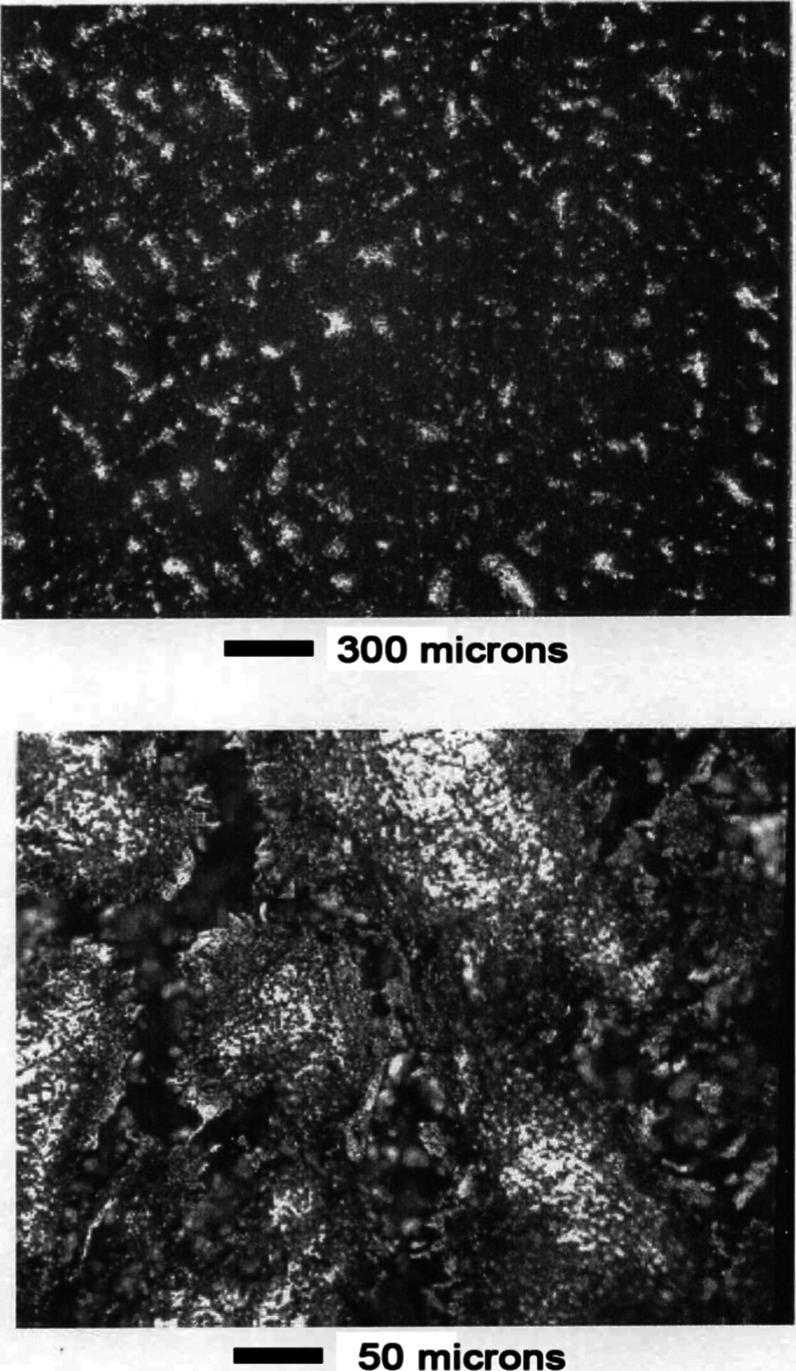
Mn–Ni Alloy #1 after leaching 13 min in 1 wt % nitric acid and 17 min in 10 wt % nitric acid.

**Figure 10:**
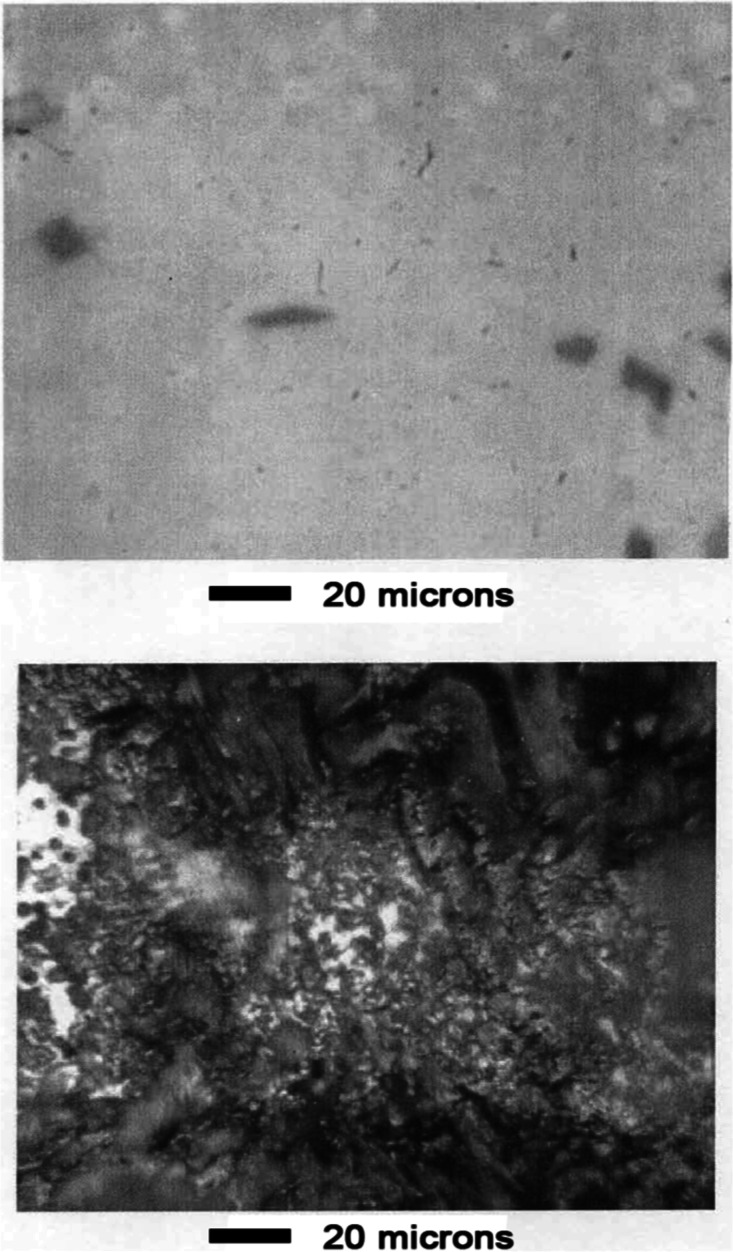
Comparison of alloy morphology before and after leaching, Mn–Ni Alloy #1.

#### 3.1.2 Leaching Tests

The alloy was crushed to a −200 mesh powder and leached in various acid solutions. The first solution tried was a 8.9 wt % acetic acid solution; however, hydrogen bubbles formed too slowly. In one case, 21 mL of hydrogen should have been produced if all of the manganese was selectively leached. After 18 h, only 0.5 mL of hydrogen had been produced.

Leaching was also tried in stronger acid solutions. When the alloy was placed in 24.5 wt % phosphoric acid, many small hydrogen bubbles formed and rose to the surface of the solution. The p*K*_a_ of phosphoric acid is 2.12 which is a stronger acid than acetic acid having p*K*_a_ = 4.75.

Repeated attempts to measure the catalytic activity of alloys leached in phosphoric acid showed that the catalysts were completely inactive for butyronitrile (BN) hydrogenation. Atomic adsorption experiments indicated that the leach solution contained large amounts of nickel. The Ni/Mn ratio of the leach solution was approximately 1 ± 0.5, indicating that manganese was not selectively leached.

Repeated attempts to prepare catalysts in acids of various strengths led to the following conclusion: manganese could not be selectively leached at any pH. Phosphoric, hydrofluoric, citric, and acetic acids were tried. If the pH was high, leaching did not occur. If the pH was lowered such that leaching did occur, both nickel and manganese dissolved. To enable selective leaching, the ratio Mn/Ni should be higher in the starting alloy.

### 3.2 Alloy # 2: Low Nickel Content

#### 3.2.1 X-ray Diffraction Results

The alloy was crushed to a powder and analyzed by X-ray diffraction. The XRD pattern matched that of alpha-manganese except that the peaks occurred at 1° lower than expected. The peaks were also a narrow doublet, with the smaller doublet occurring at higher diffraction angles. The doublets were separated by approximately 0.3°. The X-ray diffractogram is shown in Figure S1 of Supporting Information Section S1.

This diffraction pattern can be understood as follows. Nickel is slightly larger than manganese. Dissolving nickel in alpha-Mn causes the lattice constants to increase, which shifts the peaks to lower diffraction angles. The peaks are also split because two types of unit cells occur. Those which contain a nickel atom and those which do not. The unit cells containing a nickel atom are slightly larger than those which do not.

#### 3.2.2 Optical Microscopy Results

Alloy #2 was attacked by even slight water exposure, as shown in Figure 11. Spotting indicated a roughening of the surface as the manganese dissolved and the nickel relocated. Further leaching in water results in a very porous structure, as shown in Figure 12. Snake-like channels are etched out which penetrate the sample.

**Figure 11:**
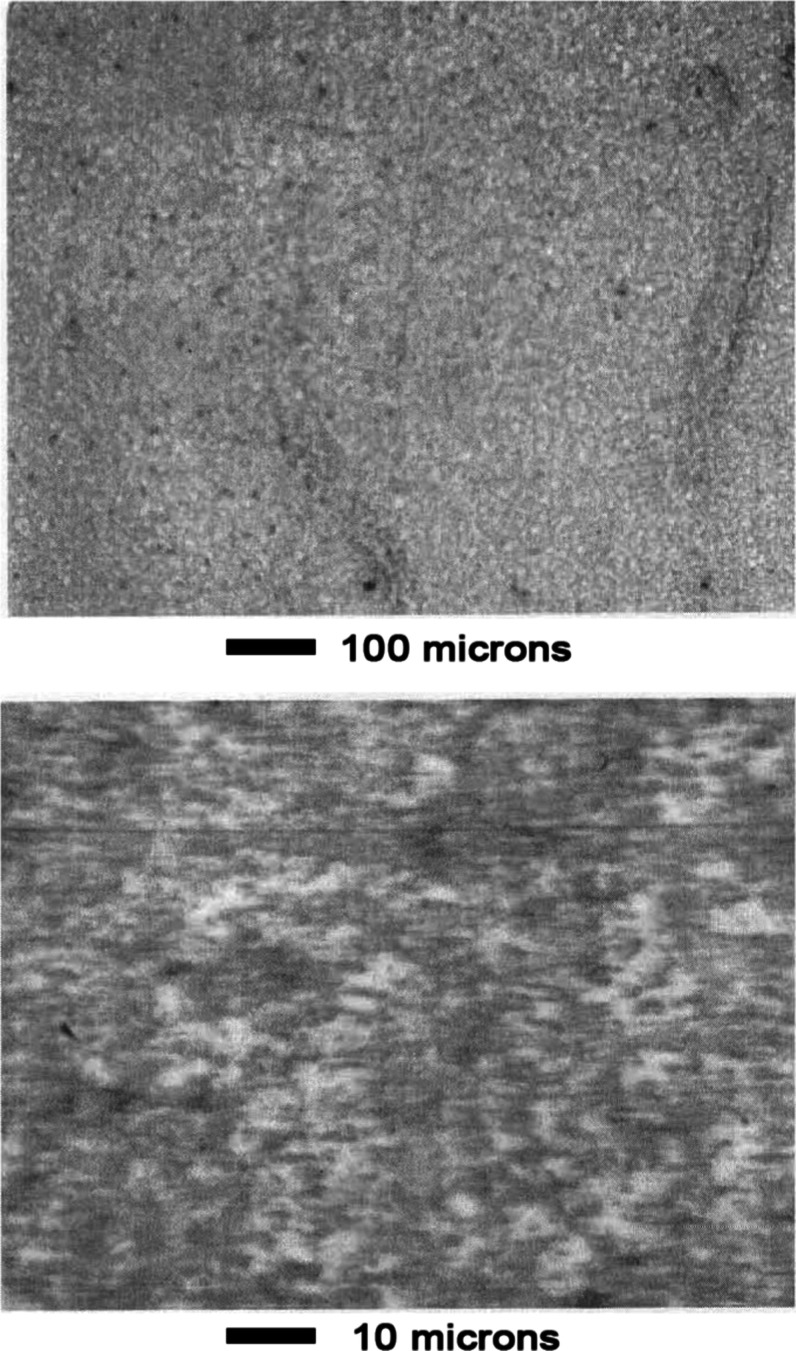
Mn–Ni Alloy #2 slight water exposure.

**Figure 12:**
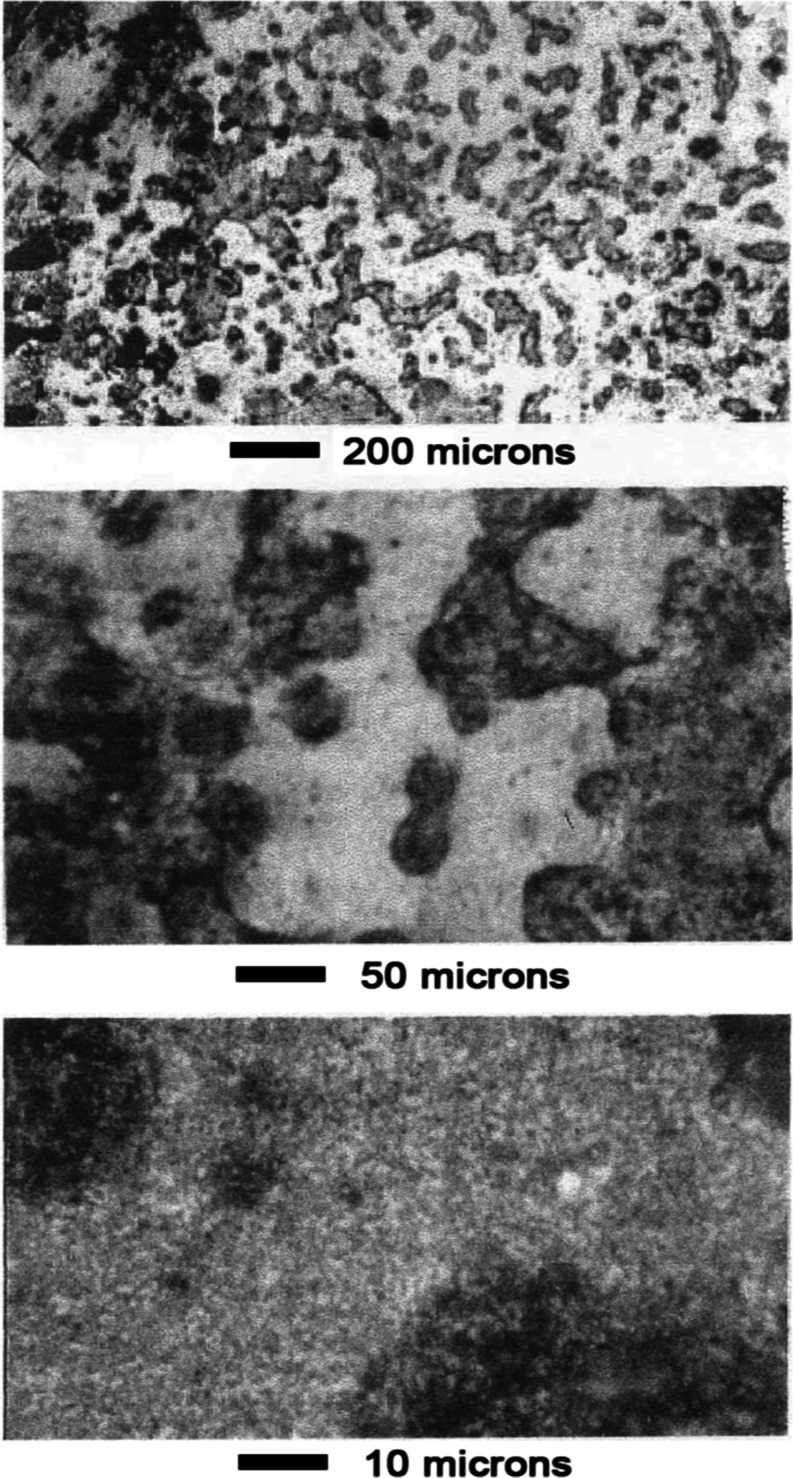
Mn–Ni Alloy #2 moderate water exposure.

After leaching for 1.5 min in 1 wt % acetic acid, the channels grew so that only a porous skeleton of nickel-rich material remained. As shown in Figure 13, the skeleton had a pumice-like structure with high porosity.

**Figure 13:**
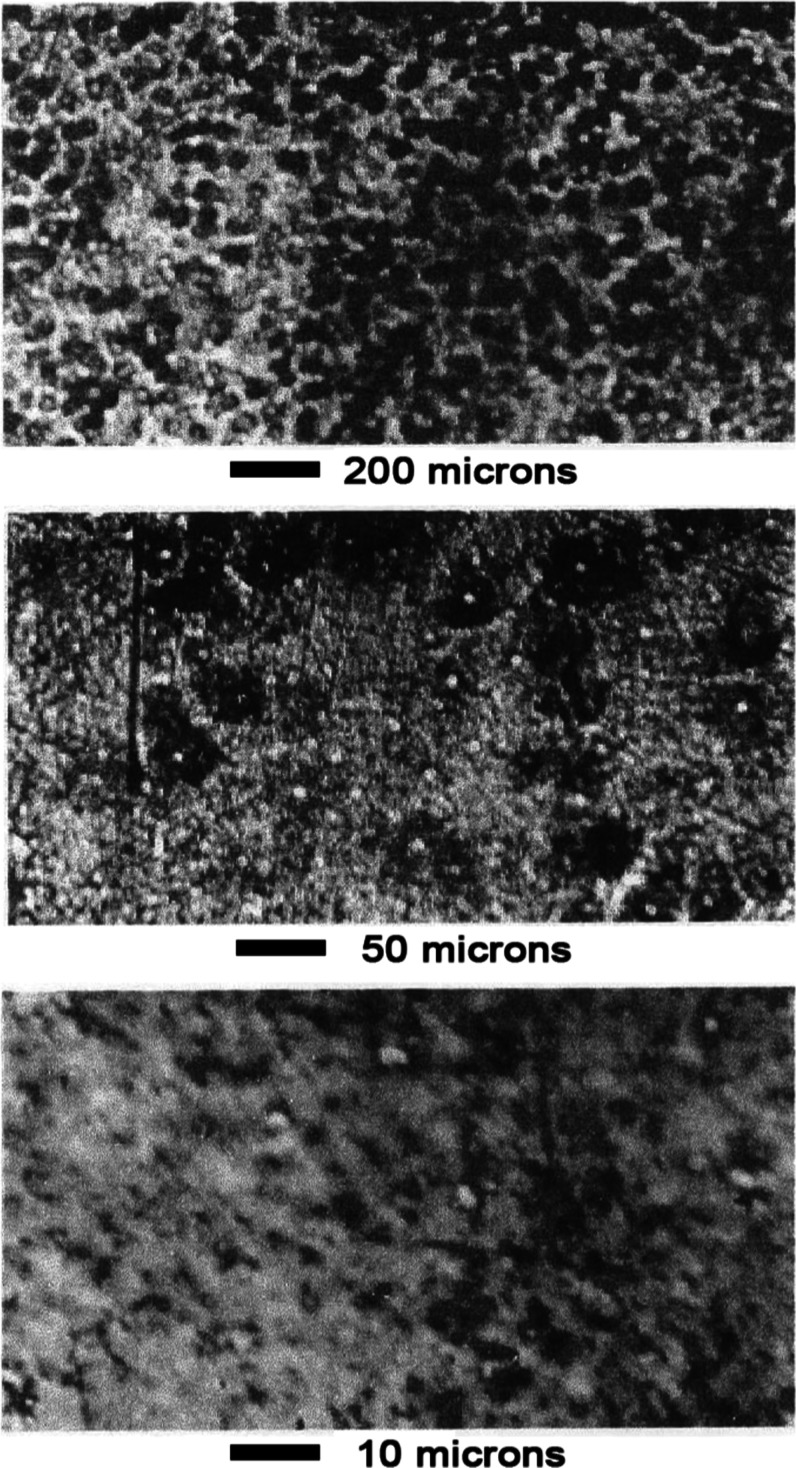
Mn–Ni Alloy #2 after leaching 1.5 min in 1 wt % acetic acid.

#### 3.2.3 Leaching and Reactivity Tests

##### 3.2.3.1 Catalyst Preparation #1

The alloy was crushed to a −200 mesh powder and then leached in excess 10 wt % acetic acid aqueous solution for 50 min at 50 °C with stirring. The catalyst was subsequently rinsed 4 times with 25 mL 1 wt % acetic acid and then 3 times with 25 mL DI water.

0.14 g catalyst preparation #1, 10 mL water, and 50 mL ethanol were placed in the reactor. The reactor was purged with hydrogen gas and then 1 mL BN was injected into the stirred glass reactor. The hydrogen gas uptake was measured and found to be 0.075 ± 0.025 mL/min. Based on this uptake, the initial rate of BN depletion was calculated to be 0.01 mmol/(g cat*min). This is a very slow rate compared to 0.12 mmol/(g cat*min) for the unpromoted Ni–Al catalyst. The catalyst was still emitting hydrogen bubbles, indicating that the leaching was incomplete.

##### 3.2.3.2 Catalyst Preparation #2

A second catalyst was prepared by adding 8.93 g alloy #2 to 78 g water, 19.5 g acetic acid, and 1.3 g NaOH. The NaOH converted some of the acetic acid to sodium acetate, thereby buffering the pH of the leaching solution around its p*K*_a_ value of 4.75. The alloy was leached at 50 °C for 27.2 h after which time it was washed 8 times in 83 mL 1 wt % acetic acid and 3 times in 83 mL DI water. During washing, fine colloidal particles became suspended in the solution. These were probably small silica particles formed from the silica impurity in the parent alloy. A small amount of catalyst preparation #2 was dissolved in concentrated nitric acid. Atomic absorption showed that the catalyst composition was 94 at. % Ni and 6 at. % Mn.

BN hydrogenation was carried out in the stirred glass reactor using 0.22 g catalyst preparation #2, 10 mL water, 50 mL methanol, and 5 mL BN. The initial hydrogen uptake was found to be approximately 0.01 mL/min. Based on this uptake, the initial rate of BN depletion was calculated to be 0.001 mmol/(g cat*min), which was 1 order of magnitude smaller than the rate of catalysts preparation #1 and 2 orders of magnitude smaller than the unpromoted Ni–Al catalyst.

##### 3.2.3.3 Catalyst Preparation #3

A third catalyst preparation was made using 4.08 g −200 mesh alloy powder. The alloy was slowly added to a 1.5 times excess 20 wt % acetic acid aqueous solution. This acid solution was buffered with sodium acetate in the ratio HAc/NaAc = 3. The alloy was leached at 70 °C for 4.75 h with moderate stirring and subsequently rinsed in acetic acid solutions of decreasing concentration. The first wash consisted of 20 mL 1 wt % acetic acid with stirring at 25 °C for 3 min. The second wash consisted of 10 mL 1 wt % acetic acid plus 10 mL DI water with stirring at 25 °C for 5 min. The third wash was the same except 1 mL acetic acid solution and 19 mL water were used. Finally, the catalyst was rinsed twice with 20 mL DI water.

Atomic absorption on catalyst preparation #3 showed that it was composed of 8.1 at. % Mn and 91.9 at. % Ni. The leaching solution was composed of 95.8 at. % Mn and 4.2 at. % Ni, which corresponds to a loss of 27% of the total nickel.

#### 3.2.4 Butyronitrile Hydrogenation

Catalyst preparation #3 was found to readily hydrogenate butyronitrile (BN). The stirred glass reactor was loaded with 0.5 g catalyst, 10 mL DI water, and 50 mL ethanol. Hydrogen flow at 1 atm pressure was initiated. After 15 min, 5 mL BN was injected into the reactor. The batch reaction was carried out at room temperature (25 °C).

Catalyst activity was similar to that of the unpromoted Ni–Al catalyst that was reported in ref 22 Figure 14 shows the concentration versus time plot. The catalyst produced 1.3 times as much DBI + DBA as the unpromoted Ni–Al catalyst. Two other byproducts were also formed in trace amounts. An intermediate “B” was formed initially and appeared to be converted to “C” which remained at the end of the reaction. The retention time for “B” was between that for ethanol and BA, while that for “C” was between BA and BN. The nature of these two compounds can be inferred from prior literature reports. Specifically, prior literature reports that ethanol first eliminates hydrogen to form acetaldehyde which condenses with butylamine and subsequently eliminates water to form an imine that is hydrogenated to *N*-ethylbutylamine.^53,62^ Almost certainly, “B” and “C” were compounds formed in this byproduct pathway involving condensation between butylamine and the ethanol solvent.

**Figure 14:**
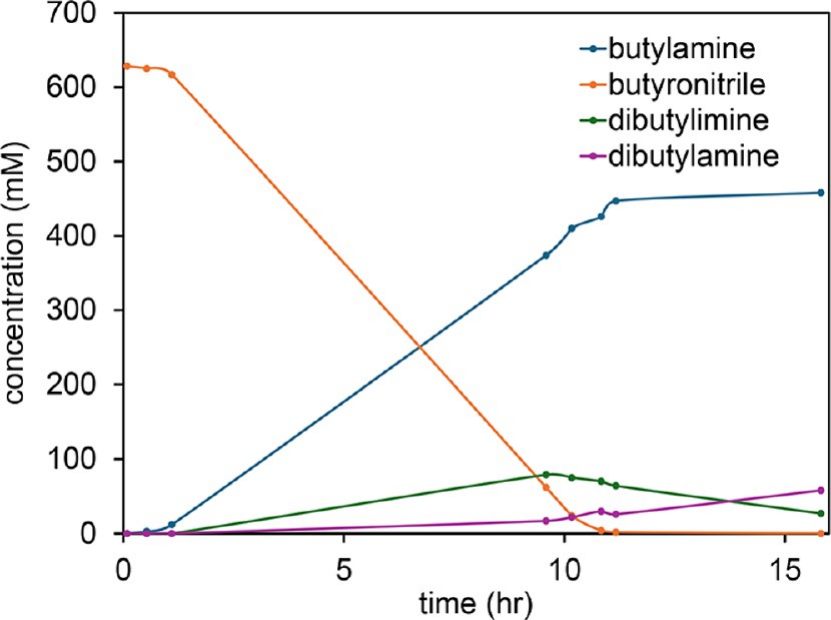
Concentration versus time profile for butyronitrile hydrogenation over Mn–Ni based sponge nickel catalyst preparation #3. Also, byproducts formed between the condensation of butylamine and ethanol solvent were produced in trace amounts (not shown).

The previous reaction solution was decanted. The catalyst was rinsed with ethanol. 50 mL ethanol and 10 mL water were added to the reactor and hydrogen flow initiated. The catalyst took up hydrogen which seemed to indicate that it was still active. Afterward, 0.50 g NaOH was added to the reactor followed by 5 mL BN. Hydrogen uptake was very slow, indicating that the catalyst was no longer active. Apparently, NaOH deactivated the catalyst.

### 3.3 Alloy #3: Medium Nickel Content

#### 3.3.1 Optical Microscopy Results

Appearance of the surface before leaching is shown in Figure 15. As shown in Figure 16, the phase structure became apparent after leaching for 1 min in 1 wt % acetic acid aqueous solution. Dark oval grains occupied most of the surface. A lighter background phase occupied the space surrounding the ovals.

**Figure 15:**
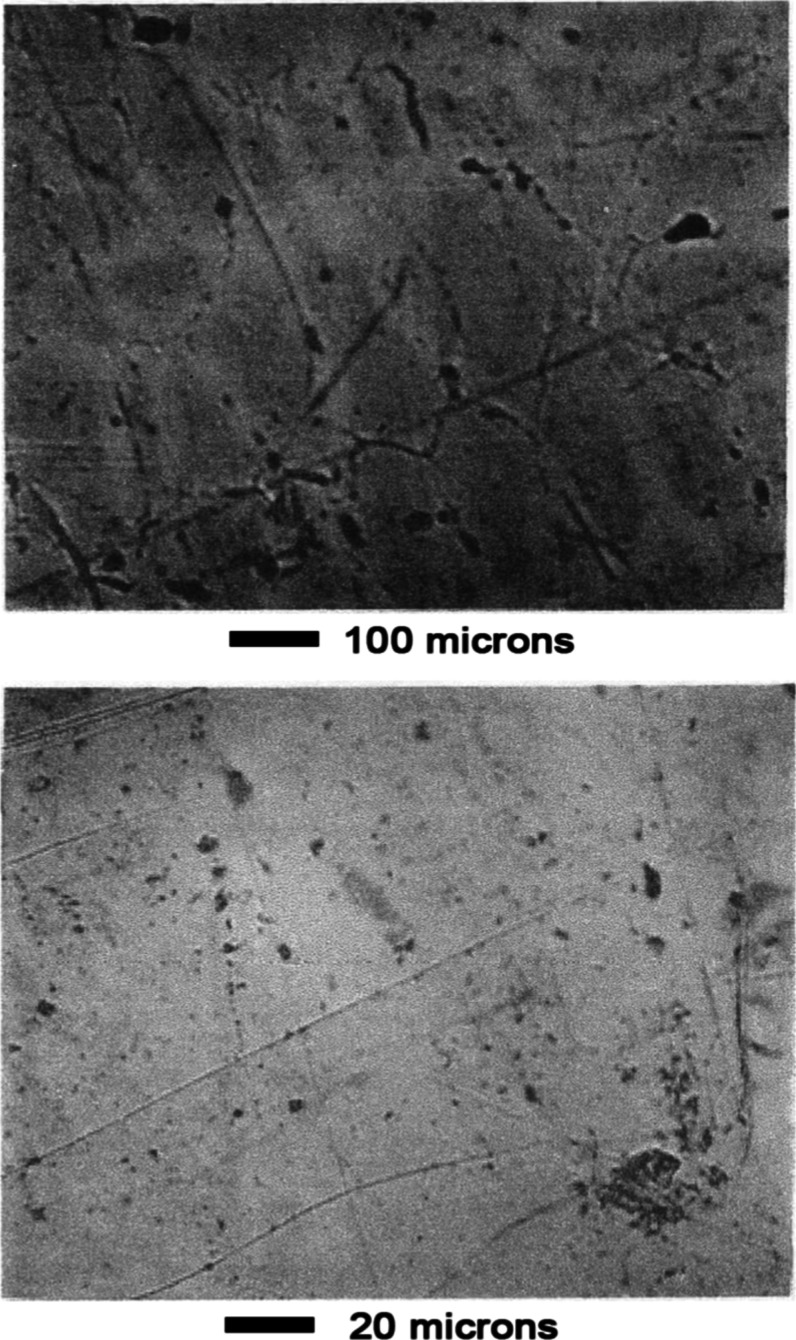
Mn–Ni Alloy #3 before leaching.

**Figure 16:**
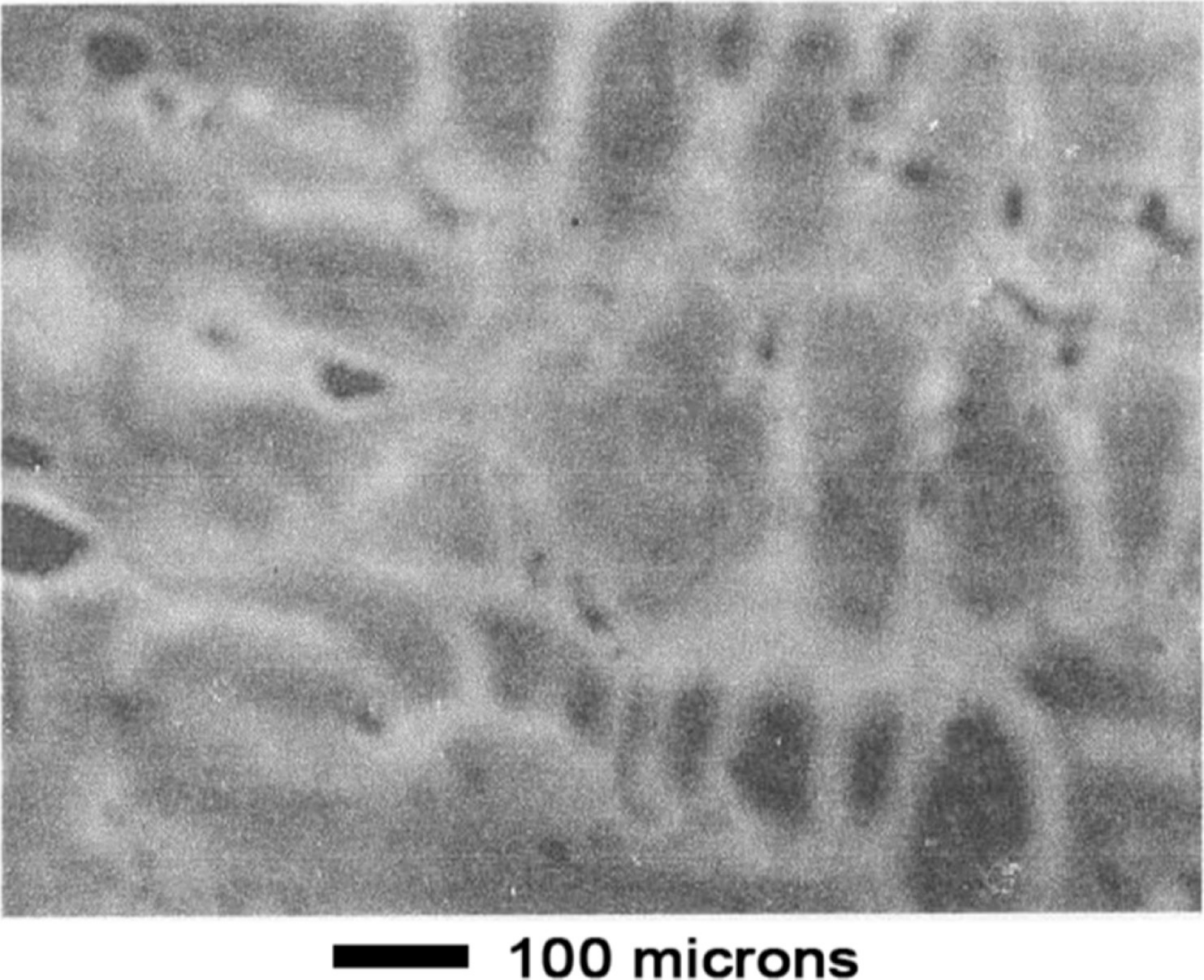
Mn–Ni Alloy #3 after leaching 1 min in 1 wt % acetic acid.

As shown in the top panel of Figure 17, contrast between the two phases was enhanced after 3 min leaching. An unusual cracking pattern was evident at high magnification as shown in the bottom panel of Figure 17. Appearance of the surface was similar after 10 min of leaching, as shown in Figure 18. The cracking pattern penetrated both phases as shown in panels a and b of Figure 19.

**Figure 17:**
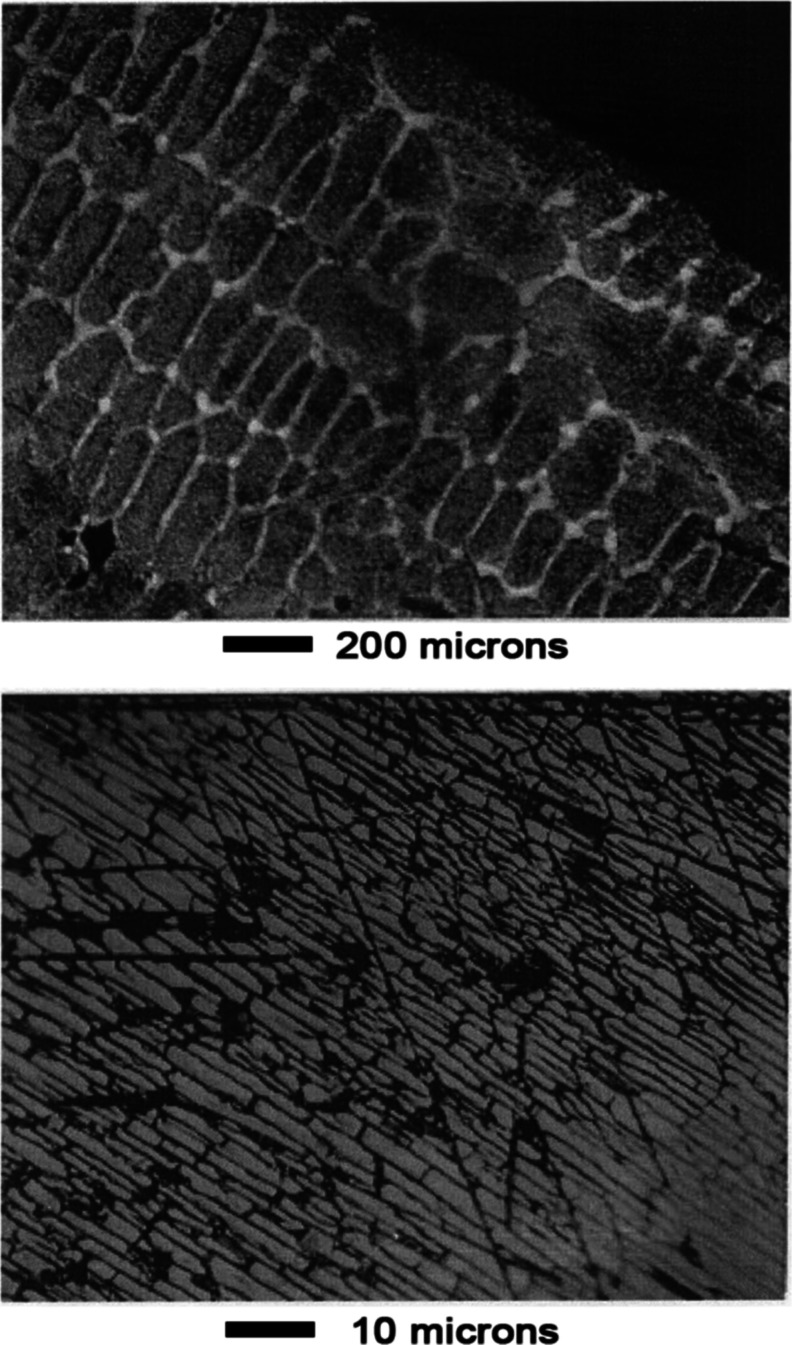
Mn–Ni Alloy #3 after leaching 3 min in 1 wt % acetic acid.

**Figure 18:**
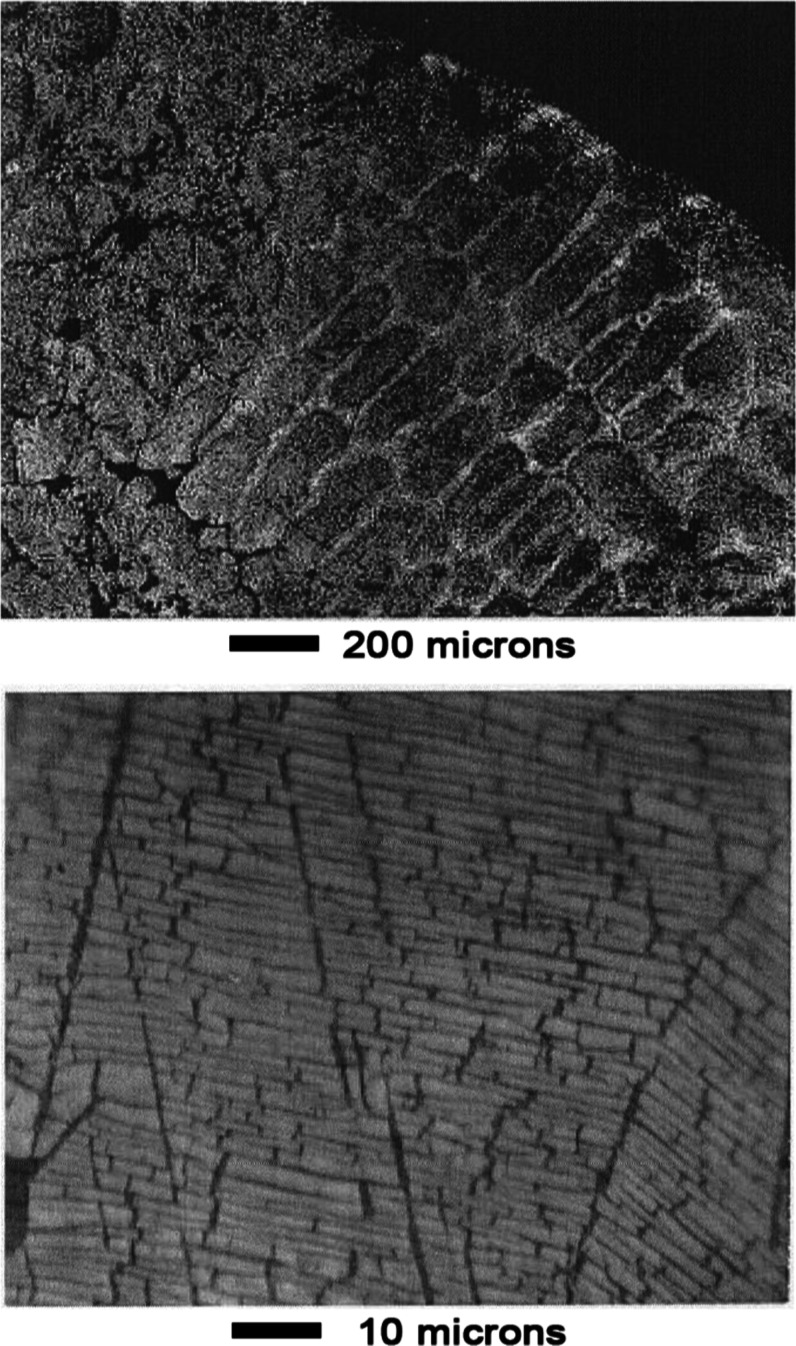
Mn–Ni Alloy # 3 after leaching 10 min in 1 wt % acetic acid.

**Figure 19:**
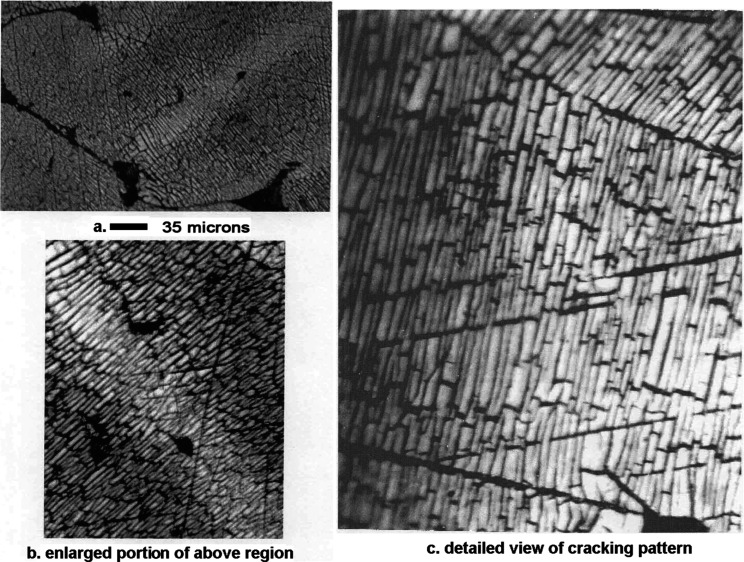
(a) Optical micrographs showing that the cracking pattern extends continuously through both alloy phases. Mn–Ni Alloy #3 after leaching 10 min in 1 wt % acetic acid. The scale shown refers to panel (a). Panels (b,c) are enlargements showing detailed views of the cracking pattern demonstrating the leached catalyst is composed of brick-like crystallites.

#### 3.3.2 Leaching Tests

Alloy #3 was not brittle and could not be crushed with a mortar and pestle, hammer, hand-turned shop vice, or a light-duty hydraulic press. This hydraulic press went up to approximately 1000 psi maximum pressure. This pressure had no discernible effect on alloy #3.

A 2.23 g billet was placed in 35 mL of 15 wt % acetic acid solution. The acetic acid solution was buffered with 1 mol sodium acetate for every 3 mol of acetic acid. Based on the manganese content of the alloy, the theoretical hydrogen evolution was calculated to be 766 mL at normal temperature and pressure (20 °C, 1 atm). After 6 days of leaching at 20 °C, 500 mL hydrogen had been evolved. This increased to 600 mL after 7 days. The leaching solution was green, indicating that some nickel had also dissolved.

Some of the dissolved nickel apparently redeposited in the form of a fine black precipitate. The billet weight of approximately 0.5 g was thus smaller than that predicted from the volume of hydrogen produced. Based on the volume of hydrogen produced, the final billet weight was predicted to be 0.95 g.

The leached billet could not be crushed with a vice or hammer. This is an extremely interesting and important result, because the metal retained its original size, shape, and an extremely high strength even though more than 50% of the atoms had been removed! The sample appeared to be a “solid metal” even though it was not. The sample was clamped in a vice and sawed into 3 smaller pieces using a hacksaw. Sawing was difficult, as the material was extremely tough. Friction during sawing caused the sample to become hot.

One of the pieces was rinsed with 10 mL 1 wt % acetic acid for 5 min and then with DI water. Liquid nitrogen adsorption was done on this sample to determine its surface area and pore structure, as described in the following subsection. A replicate run was made with similar results. Both nitrogen physisorption runs showed that the catalyst was microporous with a BET surface area of 10–12 m^2^/g.^23^ Atomic absorption showed that the catalyst contained Mn/Ni in a ratio of 1.5:1.

The other two pieces were placed back into the leaching solution for an additional 7 days. During this time, the previous catalyst structure was disintegrated. Fine particles remained which precipitated rapidly from the solution when stirred. Nitrogen physisorption on this sample was also performed as described in the following subsection.

#### 3.3.3 Surface Area and Pore Size Distribution Measurements

Surface areas and pore size distributions were extracted from N_2_ adsorption isotherms at 77 K. These liquid nitrogen adsorption and desorption isotherms were measured using the experimental procedure described in a companion article.^23^ That article reported the experimental adsorption isotherms, experimental desorption isotherms, and fitted Brunauer–Emmett–Teller (BET) and Manz–Delgass (MD) model isotherms.^23^ The resulting surface area values were: (a) 10 (BET model) and 13 (MD model) m^2^/g for Mn–Ni alloy #3 leached for 7 days at room temperature in 2.5 M acetic acid aqueous solution and (b) 32 (BET model) and 37 (MD model) m^2^/g for Mn–Ni alloy #3 leached for 14 days at room temperature in 2.5 M acetic acid aqueous solution.^23^

Supporting Information Section S2 contains Harkins–Jura (HJ) adsorption thickness t-plots and Barrett–Joyner–Halenda (BJH) pore size distribution plots for Mn–Ni alloy #3 leached at room temperature in 2.5 M acetic acid aqueous solution for 7 days and for 14 days. Each t-plot shows volume adsorbed on the *y*-axis versus adsorption thickness (computed using the Harkins–Jura equation) on the *x*-axis.^63,64^ The BJH pore size distribution is a plot of pore volume on the *y*-axis versus pore diameter on the *x*-axis as determined using BJH pore size analysis.^65,66^ As shown in Figures S3 and S4, after leaching for 7 days the material has small micropores with an average pore diameter of approximately 2 nm. As shown in Figures S5 and S6, a broad distribution of much larger pores with diameters from 2 to 50 nm occurs in the material after leaching for 14 days.

#### 3.3.4 Drilling to Produce Powder and Turnings

In an effort to produce a powder sample, the top of a drill press was first cleaned. Then a small piece of Mn–Ni alloy #3 was clamped under the drill press. The alloy was first drilled using an approximately 1/8″ drill bit. This was done multiple times in different locations on the alloy sample. An attempt to step up to an approximately 1/4″ drill bit was abandoned, because the sample became so hot that it began to smoke. The particles, turnings, and flakes were weighed and found to have a combined mass of 1.6 g. The −30 mesh (i.e., <0.0203 in.) particles were separated out using a 30 mesh screen; this fraction was found to have a mass of 0.59 g. The thin turnings were separated out from the larger flakes with a spatula; the turnings had a mass of 0.77 g. This procedure demonstrates how drilling can be used to produce small particles and thin turnings of the alloy material.

#### 3.3.5 X-ray Diffraction Results

X-ray diffractograms are shown in Figure S2 of Supporting Information Section S1. X-ray diffraction of the starting alloy showed that it was composed primarily of (γMn, Ni). Some of the peaks were shifted by 1–2° on the 2-Theta scale. This is due to the presence of nickel, which slightly altered the lattice parameters. Identification of the second phase was not possible. Either because it was present in too low of amounts to be detected, or because it is similar to the first phase and therefore has peaks in the same places. As shown in the phase diagrams of Figures 1–3, for the alloy #3 composition (∼73 at. % Mn) gamma manganese is formed at high temperature but predicted to convert to other phases below 400–700 °C. So, the observation of the (γMn, Ni) phase in the room-temperature X-ray diffraction pattern means that it persisted as a metastable phase at room temperature.

Maybe both phases in the microstructure are variations of the same crystal pattern. The difference between the two could be that one contains more nickel than the other. One may be ordered and the other disordered or both may be disordered phases? This hypothesis is based on the X-ray diffraction pattern and optical microscopy which shows that both phases give rise to similar cracking patterns. The cracks extend continuously from one phase to the next, potentially indicating the crystal plane orientations are the same in both phases? Further research is required to answer these questions.

X-ray diffraction on a sample leached in acetic acid gave a spectrum similar to an unleached one. This could mean either that the resulting sponge microstructure is a nanoporous (γMn, Ni) phase or that the leaching time was too short and the original nonporous (γMn, Ni) phase still remained in the leached sample. Further research is required to resolve this question.

## 4 Discussion: Potential Applications of This Nanoporous Crystalline Alloy

We suggest the term “nanoporous crystalline alloy” (NCA) be used for any material that meets the following criteria.

1. Has been derived by leaching an alloy of two or more metals.
2. Has a surface area greater than 1 m^2^/g.
3. Has a crystalline structure in which the majority of surface area is located in nanopores inside the crystals as opposed to larger pores between the crystals.
4. Has sufficient strength to hold its original overall shape.

The Mn–Ni alloy #3 sample leached for 7 days at room temperature in 15 wt % acetic acid is a material of this type. It contained nanopores yet retained its overall shape, appearance, and apparent strength.

Panel c of Figure 19 shows an enlarged view of the microstructure after leaching 10 min in 1 wt % acetic acid. The crystallites shown are approximately 2–3 × 5–15 μm in size. Each crystallite contains many nanopores that are too small to be seen by optical microscopy. The overall surface area of the material was 10–13 m^2^/ g.

What are some possible uses of this material? Due to its high strength and low apparent density, it should find applications in industries requiring strong lightweight materials. This NCA material has an apparent overall density that is 31 ± 0.9% that of the original alloy: 0.31 × 8 g/cm^3^ = 2.5 ± 0.8 g/cm^3^. Table 3 compares its density to that of several metal elements. Pure aluminum is soft and lacks strength, but it can be alloyed to produce stronger materials. Titanium is 60% heavier than aluminum, but twice as strong. Titanium is expensive, but it is as strong as steel with 45% less weight. The Mn–Ni NCA material has a density comparable to that of aluminum and is a strong material.

**Table 3:** Density of Metallic Elements at 25 °C

metal	density^a^ (g cm^–3^)	strength	comments
light alkali metals	0.534 (Li), 0.97 (Na), 0.89 (K)	extremely soft, lack strength	too reactive
light alkaline earths	1.85 (Be), 1.74 (Mg), 1.54 (Ca), 2.64 (Sr)	soft, ductile	alloys used in space applications
Al	2.70	pure Al is soft, ductile, and lacks strength	strong, lightweight materials can be made from aluminum alloys
Mn–Ni NCA	2.5 ± 0.8	strong	porous
Sc	2.99	soft	easily oxidized, rare earth element
Ti	4.506	strong	corrosion resistant, used in space applications
Fe	7.87	alloys strong	inexpensive, used to make steel
Ni	8.90	hard, ductile	corrosion-resistant due to forming a protective oxide layer
other 3d transition elements	6.0 (V), 7.15 (Cr), 7.3 (Mn), 8.86 (Co), 8.96 (Cu), 7.134 (Zn)	varies	these 3d transition metals have a density of 6–9

a The density of the Mn–Ni nanoporous metal is from this work. Densities of the pure metals were taken from pp. 216–217 of Section 12 of ref 77.

Metal foams having a gas-filled cellular structure are strong, lightweight, and excellent at adsorbing impact energy.^67–72^ Dealloying procedures are an approach (but not the only approach) to preparing lightweight nanoporous metals.^25–29,73^ Strong lightweight materials are especially needed in the aerospace industry.^74–76^

If a mixture of large and small molecules is passed over the nanoporous metal material, the larger molecules could adsorb only in the larger diameter pores while the smaller molecules could adsorb in the small nanopores. Also, smaller molecules should be able to diffuse more easily through the material, because they can fit through small channels that are impassable to large molecules. For smaller molecules, a thin sheet of the Mn–Ni NCA material could potentially act as a controlled release barrier. Small molecules could diffuse through the pores of the material to go from one side to the other. A mixture of small and large molecules could potentially be separated using these size exclusion effects.

Future work is needed to precisely quantify the molecular size that defines a molecule small enough to adsorb and diffuse through this material’s pores versus a molecule that is too large to do so. Since the N_2_ molecule adsorbed in this material’s pores (as shown in Figures S3 and S4), it is clear that N_2_ and smaller molecules can fit into the pores. However, it is not yet known whether a molecule the size of naphthalene could readily fit into this material’s pores. We recommend that future experiments be performed with molecules of various sizes to quantify size exclusion effects in this material.

Over half of the volume of the Mn–Ni NCA material is occupied by a gas (such as air) or a liquid (such as water). Thus, under some conditions, this material might find applications as a metallic sponge that takes up liquids due to capillary forces.

## 5 Conclusions

Several Mn–Ni alloys were synthesized and leached in concentrated aqueous acetic acid solutions. These alloys were characterized using optical microscopy, electron microprobe analysis, and X-ray diffraction. The leached materials were characterized using nitrogen physisorption at 77 K, optical microscopy, and elemental composition analysis.

Mn–Ni alloy #1 contained ∼57 at. % Mn and was only selectively leachable near its surface. When leached in strong and moderate acids, both the Mn and Ni dissolved. When leaching was attempted in weak acids such as acetic acid, only the near-surface Mn was leached with deeper Mn atoms being unleachable. We concluded that alloys containing higher Mn/Ni ratios are needed to enhance leachability.

Mn–Ni alloy #2 contained ∼86 at. % Mn and was easily crushed to form a powder. This powder was readily leachable in concentrated aqueous acetic acid. The resulting sponge nickel catalyst had a pumice-like microstructure. This catalyst was used to hydrogenate butyronitrile to butylamine (with some formation of dibutylamine byproduct) in ethanol–water solvent at room temperature under flowing hydrogen gas. This Mn–Ni-based catalyst gave rise to higher organic compound condensation rates than the Al–Ni-based catalyst. Accordingly, we recommend that these types of Mn–Ni-based sponge catalysts be further investigated for potential applications in organic condensation reactions.

Mn–Ni alloy #3 contained ∼73 at. % Mn. This alloy was extremely strong and nonbrittle. The alloy would not break even when hammered vigorously and when placed in a hydraulic vice under approximately 1000 psi pressure. When leached in concentrated aqueous acetic acid solution for up to 7 days, this material formed a brick-like microstructure. These bricks were nanoporous with a surface area of ∼10 m^2^/g. After being leached for 7 days, this porous material was extremely strong, lightweight, nonbrittle, and had an overall density slightly less than pure aluminum metal. The leached billet could not be crushed with a vice or hammer. This is a very interesting result because the metal retained its original size, shape, and an extremely high strength even though more than 50% of the atoms had been removed! If alloy #3 was overleached (e.g., leached for 14 days in concentrated aqueous acetic acid solution), then the strong brick-like nanoporous structure collapsed and formed a powdery sponge nickel material with large pores.

Further study is needed to develop applications for these and related materials. Compositions near alloy #3 could find applications as lightweight, low-density, strong structural materials postleaching. We recommend additional research be performed to measure the mechanical properties and impact resistance of the Mn–Ni NCA material.

This work demonstrates that new sponge-type porous metals and catalysts can produce unexpected results. There remains a whole host of possible materials that have yet to be made or characterized. As indicated by the Mn–Co phase diagram (see refs 78 and 79 ), making sponge cobalt from a Mn–Co alloy is a potential extension of this work.

## Supplementary Material



## Notes

X-ray diffractograms for alloys #2 and 3; adsorption thickness and pore size distribution plots for alloy #3 leached in concentrated acetic acid aqueous solution; safety considerations (PDF)

## ⊥

Chemical & Materials Engineering, New Mexico State University, Las Cruces, NM 88003

## #

Deceased April 7, 2013.

## ¶

Deceased September 13, 2014.

## ∇

Deceased August 17, 2021.
