# Supplementary material for: Porous Metals Formed by Leaching Mn–Ni Alloys
Source: ACS Omega. 2026 Apr 27;11(18):26824–40. doi: 10.1021/acsomega.5c13660 (PMC13177224; doi:10.1021/acsomega.5c13660)
Supplement: Supplementary file 1 [file ao5c13660_si_001.pdf]

Electronic Supporting Information for

Porous Metals formed by Leaching Mn-Ni Alloys

Thomas A. Manz, David R. Gaskell, Kevin P. Trumble, Zhufang Liu, David Roberts, Carl Hager, Sourav K. Sengupta, Theodore A. Koch, and W. Nicholas Delgass
*corresponding author email: thomasamanz@gmail.com

Contents

X-ray diffractograms

Adsorption thickness and pore size distribution plots for alloy #3 leached in concentrated acetic acid aqueous solution

Safety considerations

S1. X-ray diffractograms

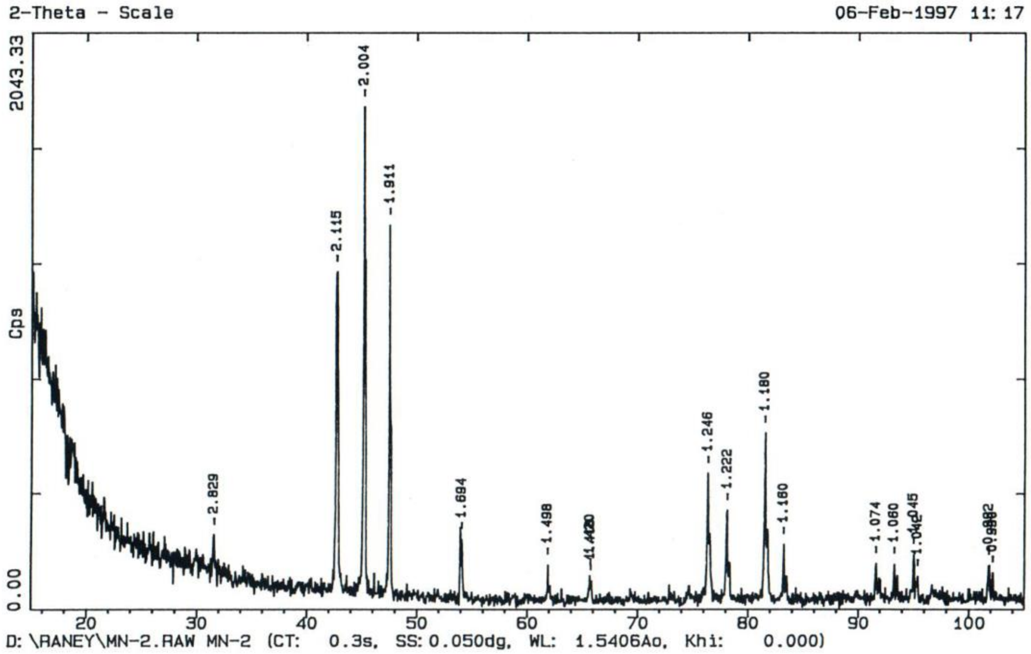

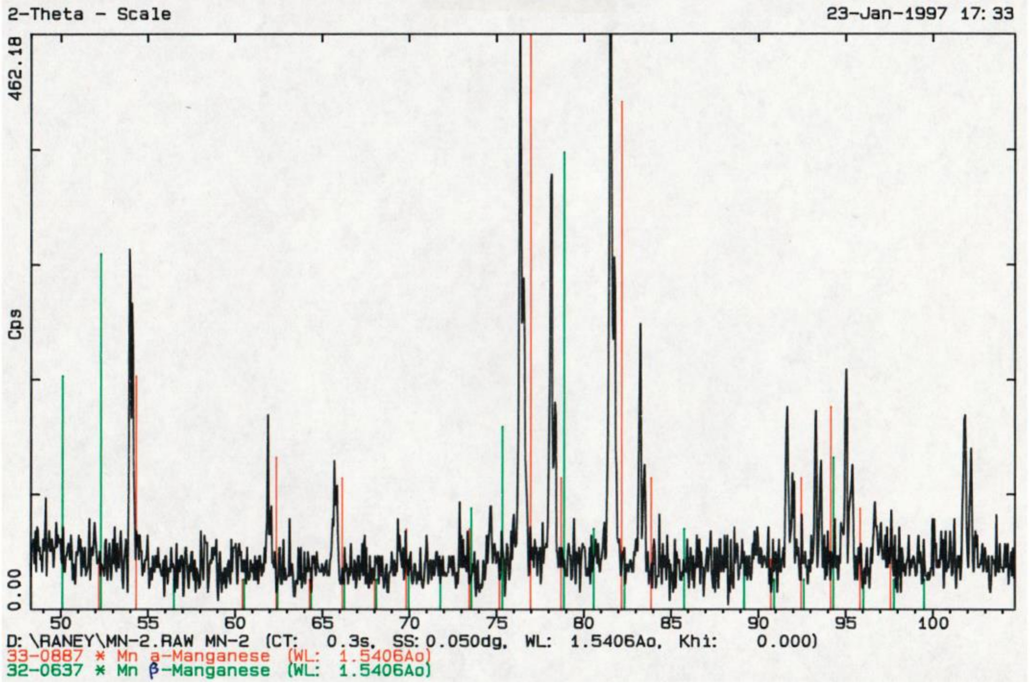

Figure S1: X-ray diffractogram of Mn-Ni alloy #2 before leaching. As shown in the top panel, the number above each peak is the interplanar spacing in angstroms. The bottom panel compares the sample's XRD pattern to that of α-Mn and β-Mn. The pattern matches that of α-Mn, except the peak positions are slightly shifted due to the presence of Ni atoms in the lattice structure. Specifically, the nickel atoms in the Mn-Ni alloy #2 caused the lattice vector lengths to slightly decrease compared to the pure α-Mn reference phase, and by Bragg's Diffraction Law this slightly smaller unit cell size caused each XRD peak for the Mn-Ni alloy #2 to shift to slightly larger 2θ value compared to the pure α-Mn reference phase.

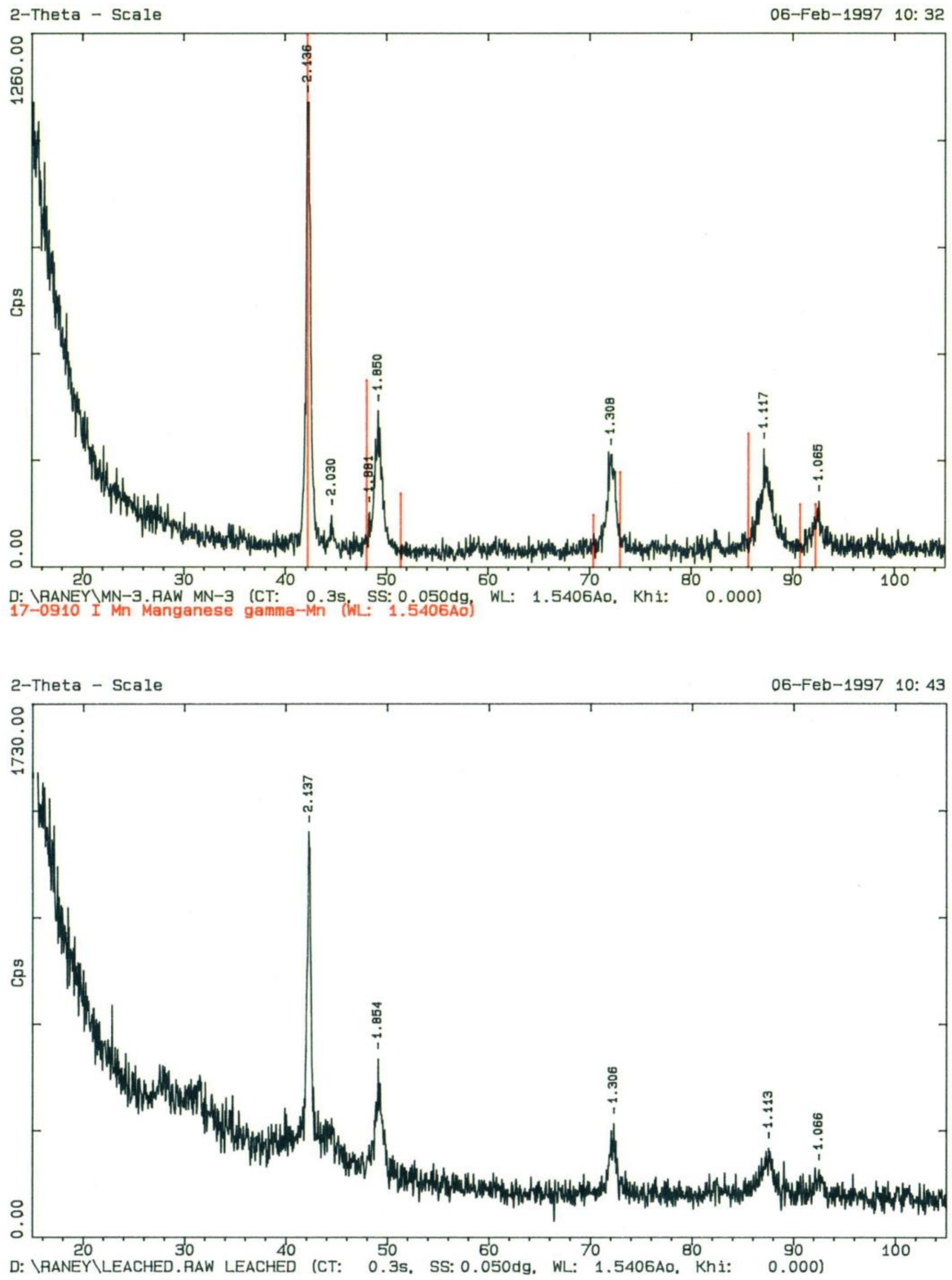

Figure S2: X-ray diffractogram of Mn-Ni alloy #3 before leaching (top panel) and after leaching in concentrated acetic acid aqueous solution (bottom panel). The number above each peak is the interplanar spacing in angstroms. The top panel compares the sample's XRD pattern to that of γ-Mn. The pattern matches that of γ-Mn, except the peak positions are slightly shifted due to the presence of Ni atoms in the lattice structure. The pattern after leaching (bottom panel) still contains some γ-Mn phase.

S2. Adsorption thickness and pore size distribution plots for alloy #3 leached in concentrated acetic acid aqueous solution

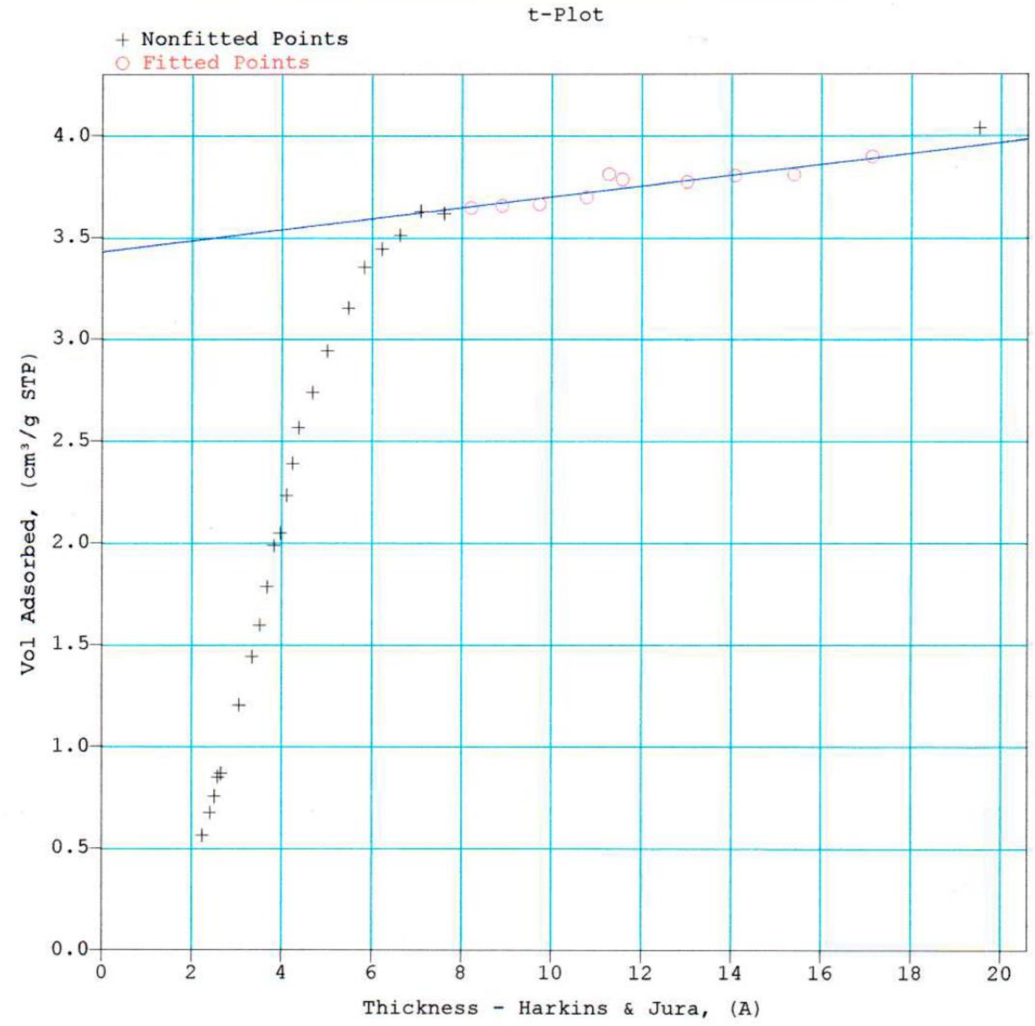

Figure S3: Harkins-Jura (HJ) adsorption thickness t-plot for Mn-Ni alloy #3 leached 7 days in concentrated acetic acid aqueous solution.

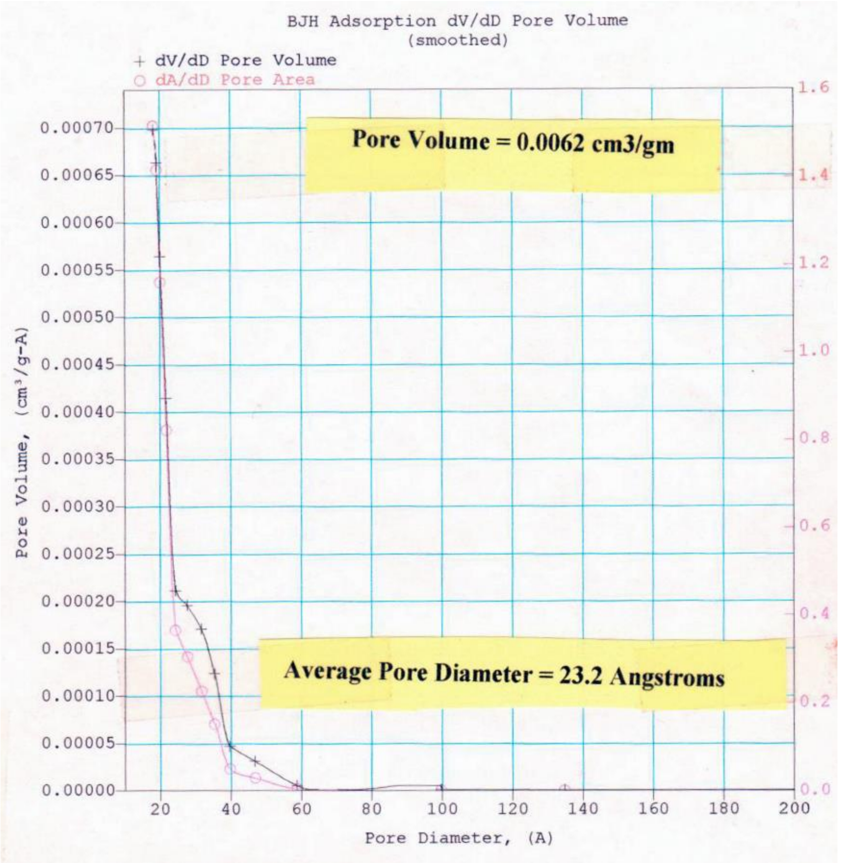

Figure S4: Barrett-Joyner-Halenda (BJH) pore size distribution for Mn-Ni alloy #3 leached 7 days in concentrated acetic acid aqueous solution.

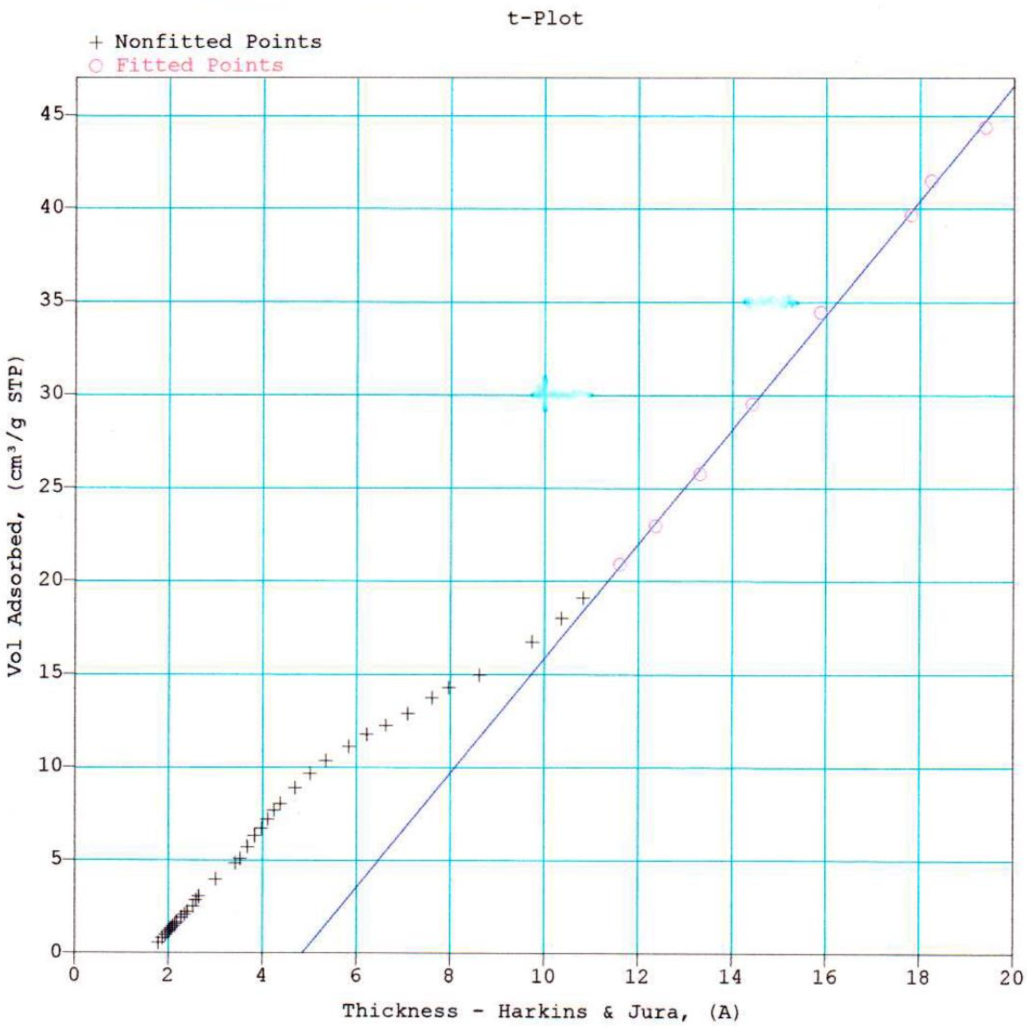

Figure S5: Harkins-Jura (HJ) adsorption thickness t-plot for Mn-Ni alloy #3 leached 14 days in concentrated acetic acid aqueous solution.

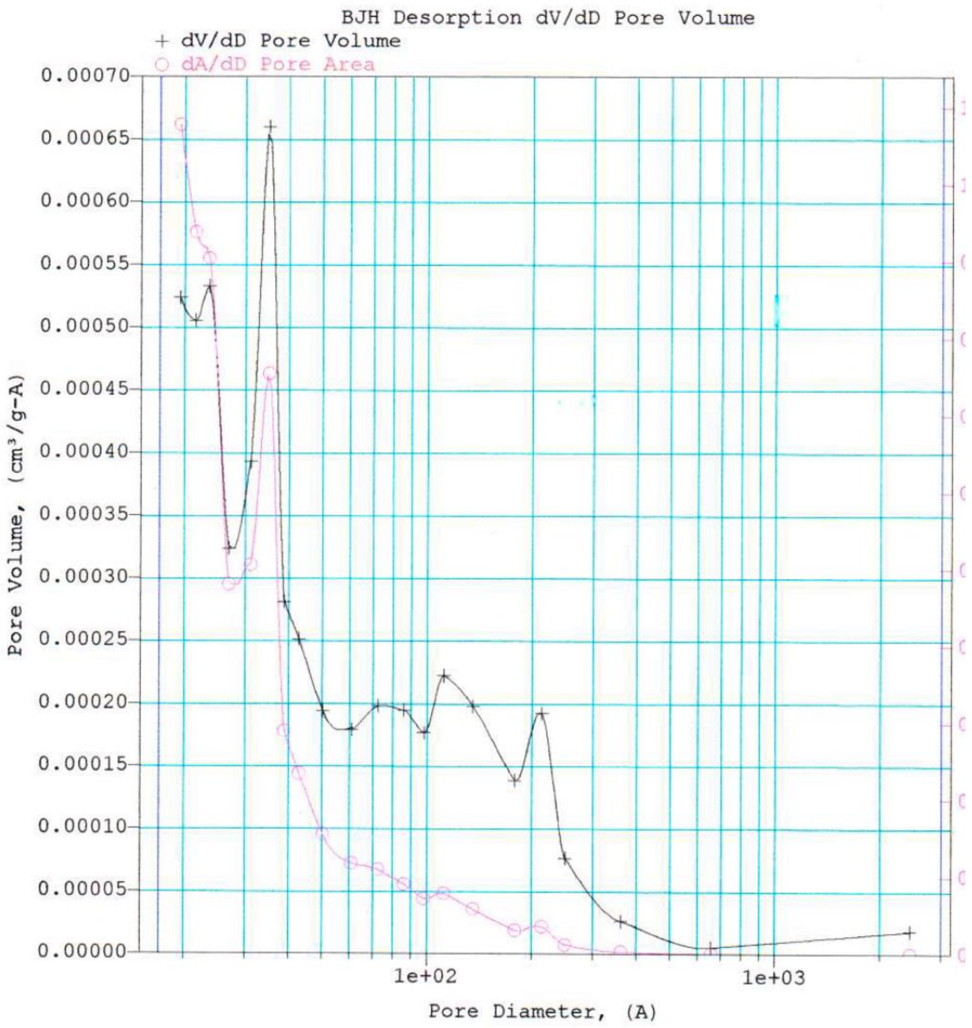

Figure S6: Barrett-Joyner-Halenda (BJH) pore size distribution for Mn-Ni alloy #3 leached 14 days in concentrated acetic acid aqueous solution.

S3. Safety considerations

When heated to high temperatures, some of the manganese evaporates during preparation of the Mn-Ni alloys. (Manganese metal has a melting point of 1517 K and a boiling point of 2392 K.[S1-S2]) The heated manganese is also prone to oxidation, so it is important to conduct such heating under an inert atmosphere. In this work, the Mn-Ni alloys were prepared in a tube furnace under flowing inert gas (e.g., helium).

During leaching, hydrogen gas is emitted. To prevent the build-up of flammable hydrogen gas, it is important to perform leaching and reactions experiments with adequate ventilation (e.g., in a fume hood). To minimize the initial surge of hydrogen gas produced, the alloy powder was added in small increments over a period of time to the leaching solution. Precautions should also be taken to avoid ignition sources (e.g., sparks) near the hydrogen gas. Hydrogen gas should not be introduced into a closed vessel filled with air. The vessel should be purged with an inert gas before hydrogen is introduced. The reactor used in this study was set-up to be purged with nitrogen gas.

Because of their high volatility and flammability, solvents and reaction chemicals were used in a fume hood and stored in sealed containers when not in use. In this study, several toxic chemicals were used that have the ability to be absorbed directly through the skin. Also, this study involved working with strong acids and strong bases. When working with toxic liquids, strong acids, and strong bases, it is extremely important to wear appropriate protective gloves, safety goggles, and a lab coat. Even with gloves on, direct contact with liquid chemicals was avoided. The Safety Data Sheet for each chemical should be consulted for specific hazards and precautions associated with each particular chemical. The spent chemicals were sunsetted by the environmental and safety department.

Nickel and its compounds are considered carcinogenic, so it is important to take appropriate safety precautions to limit inhalation of these materials, their dusts, and their fumes.

Great care must be used when handling fine metal powders. The powder can become airborne and inhaled. The risk of the powder becoming airborne is reduced when it is kept under water. The Mn-Ni alloys #1 and #2 were crushed under water to reduce powder fly-off.

Safe handling of compressed gas cylinders is important. A gas cylinder must always be secured and never moved unless properly capped. The cylinder valve should always be closed when connecting and disconnecting gas lines. After connecting gas lines, the pipe connections must be carefully checked for leaks and adjusted to fix them.

In addition to the above-mentioned specific safety considerations, all appropriate laboratory safety precautions, including wearing appropriate personal protective equipment (PPE), should be practiced when handling these materials and performing these types of experiments.

Reference:

S1. Nesmeyanov, A.N. *Vapor Pressure of the Chemical Elements*; Gary, R., Ed.; Elsevier: Amsterdam, 1963, pp. 432-443.

S2. Brandes, E.A.; Flint, R.F. Properties of Manganese. *Bull. Alloy Phase Diagr.* **1981** *2*, 113-114.
